# Parasites Affect Food Web Structure Primarily through Increased Diversity and Complexity

**DOI:** 10.1371/journal.pbio.1001579

**Published:** 2013-06-11

**Authors:** Jennifer A. Dunne, Kevin D. Lafferty, Andrew P. Dobson, Ryan F. Hechinger, Armand M. Kuris, Neo D. Martinez, John P. McLaughlin, Kim N. Mouritsen, Robert Poulin, Karsten Reise, Daniel B. Stouffer, David W. Thieltges, Richard J. Williams, Claus Dieter Zander

**Affiliations:** 1 Santa Fe Institute, Santa Fe, New Mexico, United States of America; 2 Pacific Ecoinformatics and Computational Ecology Lab, Berkeley, California, United States of America; 3 Western Ecological Research Center, United States Geological Survey, c/o Marine Science Institute, University of California, Santa Barbara, California, United States of America; 4 Ecology and Evolutionary Biology, Princeton University, Princeton, New Jersey, United States of America; 5 Ecology, Evolution and Marine Biology, University of California, Santa Barbara, California, United States of America; 6 Department of Biological Sciences, Aarhus University, Aarhus, Denmark; 7 Department of Zoology, University of Otago, Dunedin, New Zealand; 8 Alfred Wegener Institute for Polar and Marine Research, List, Germany; 9 Integrative Ecology Group, Estación Biológica de Doñana, Sevilla, Spain; 10 Royal Netherlands Institute for Sea Research, Den Burg, The Netherlands; 11 Microsoft Research, Cambridge, United Kingdom; 12 Biozentrum Grindel und Zoologisches Museum, Universität Hamburg, Hamburg, Germany; Centre National de la Recherche Scientifique, France

## Abstract

Comparative research on food web structure has revealed generalities in trophic organization, produced simple models, and allowed assessment of robustness to species loss. These studies have mostly focused on free-living species. Recent research has suggested that inclusion of parasites alters structure. We assess whether such changes in network structure result from unique roles and traits of parasites or from changes to diversity and complexity. We analyzed seven highly resolved food webs that include metazoan parasite data. Our analyses show that adding parasites usually increases link density and connectance (simple measures of complexity), particularly when including concomitant links (links from predators to parasites of their prey). However, we clarify prior claims that parasites “dominate” food web links. Although parasites can be involved in a majority of links, in most cases classic predation links outnumber classic parasitism links. Regarding network structure, observed changes in degree distributions, 14 commonly studied metrics, and link probabilities are consistent with scale-dependent changes in structure associated with changes in diversity and complexity. Parasite and free-living species thus have similar effects on these aspects of structure. However, two changes point to unique roles of parasites. First, adding parasites and concomitant links strongly alters the frequency of most motifs of interactions among three taxa, reflecting parasites' roles as resources for predators of their hosts, driven by trophic intimacy with their hosts. Second, compared to free-living consumers, many parasites' feeding niches appear broader and less contiguous, which may reflect complex life cycles and small body sizes. This study provides new insights about generic versus unique impacts of parasites on food web structure, extends the generality of food web theory, gives a more rigorous framework for assessing the impact of any species on trophic organization, identifies limitations of current food web models, and provides direction for future structural and dynamical models.

## Abstract 2

Parasites primarily affect food web structure through changes to diversity and complexity. However, compared to free-living species, their life-history traits lead to more complex feeding niches and altered motifs.

## Author Summary

Food webs are networks of feeding interactions among species. Although parasites comprise a large proportion of species diversity, they have generally been underrepresented in food web data and analyses. Previous analyses of the few datasets that contain parasites have indicated that their inclusion alters network structure. However, it is unclear whether those alterations were a result of unique roles that parasites play, or resulted from the changes in diversity and complexity that would happen when any type of species is added to a food web. In this study, we analyzed many aspects of the network structure of seven highly resolved coastal estuary or marine food webs with parasites. In most cases, we found that including parasites in the analysis results in generic changes to food web structure that would be expected with increased diversity and complexity. However, in terms of specific patterns of links in the food web (“motifs”) and the breadth and contiguity of feeding niches, parasites do appear to alter structure in ways that result from unique traits—in particular, their close physical intimacy with their hosts, their complex life cycles, and their small body sizes. Thus, this study disentangles unique from generic effects of parasites on food web organization, providing better understanding of similarities and differences between parasites and free-living species in their roles as consumers and resources.

## Introduction

Ecological network research is a powerful framework for assessing ecosystem organization, dynamics, stability, and function, topics that are central to ecology [1]–[7]. For example, comparative studies of food web structure have revealed regularities in how consumer–resource interactions (Box 1) among species are organized [8]–[12], produced successful simple models to characterize such structure [13]–[16], and supported research on the robustness (Box 1) of food webs to species loss [17]–[20]. These and other insights, however, have been largely based on analyses of interactions among free-living species, and have generally neglected parasites. Parasites comprise a significant part of the earth's biodiversity [21], can achieve substantial biomass in some ecosystems [22], can have similar abundance and productivity to free-living species of comparable body size and trophic level [23], and likely extend the generality of the metabolic theory of ecology [24]. Further, in terms of their trophic relations, parasites have consumer–resource body-size ratios inverse to those of most free-living predators [23], which enhances their ability to regulate host species abundances [25]; they have durable physical intimacy with their hosts [26]; they often have complex life cycles, sometimes requiring multiple phylogenetically distant hosts of widely varying body sizes over a lifetime [27]; they may have different patterns of trophic specialization than free-living predators [28]; they may differentially associate with hosts in different topological positions in food webs [29],[30]; and their manipulation of hosts can reorganize communities and alter ecosystem function [31]. These and other ecological factors might alter how parasites fit into, and affect the structure of, food webs compared to free-living organisms. For example, although some parasites appear to be trophic generalists (Box 1), when their hosts are aggregated over their whole life cycle, they are actually temporal serial specialists (Box 1), with particular hosts at particular life stages [32]. Taking this into account increases the likelihood that primary species loss will lead to secondary extinction of such parasites and also decreases the robustness of the food web in question [32]–[35]. In general, the great diversity and unique habits and roles of parasites suggest that their explicit inclusion in food webs may alter our understanding of species coexistence and ecosystem structure, stability, and function [35]–[40].

**Box 1. Glossary**

**Complexity:** In most food web studies, complexity refers to simple relationships between the number of feeding links *L* and the number of taxa *S* in a web, particularly link density (*L/S*) and connectance (*C*) (Table 1).

**Consumer–resource interaction:** An interaction whereby an individual of species A (the consumer) feeds on an individual of species B (the resource), resulting in a transfer of biomass from B to A. It includes all types of feeding interactions, such as predator–prey, herbivore–plant, parasite–host, and detritivore–detritus.

**Concomitant links:** Trophic links from a free-living consumer to the parasites of its resources [38],[45],[66].

**Degree distribution (cumulative):** The proportions of species *P*(*k*) that have *k* or more trophic links in a food web [8],[10]. This study focuses on the resource distribution, the numbers of links to resource taxa (i.e., numbers of resource taxa per consumer), and the consumer distribution, the numbers of links to consumer taxa (i.e., numbers of consumer taxa per resource). The resource distribution reflects the balance of specialists and generalists in a food web, while the consumer distribution reflects the balance of invulnerable and vulnerable species in a food web.

**Diversity:** In most food web studies, diversity is measured as species richness *S*, the number of taxa (nodes) in the web.

**Food web:** The network of feeding interactions among co-occurring taxa in a particular habitat.

**Generalist:** A consumer taxon that feeds on multiple resource taxa.

**Generality:** How many resource taxa a consumer taxon has.

**MaxEnt model:** A model that generates the least biased probability distributions by maximizing the information entropy for a system after applying information-containing constraints [71]. In the current study, it is applied to degree distributions to provide a null expectation for the shape of food web consumer and resource distributions [62].

**Motifs:** In this study, the 13 unique link patterns (including both single- and bidirectional links) that can occur among three taxa, excluding cannibalistic links. The frequency of a motif in an empirical web is compared to its frequency in an ensemble of randomized webs to determine whether the motif is under- or overrepresented in the empirical web or a set of model webs [11].

**Network structure:** The patterns of how links are arranged among nodes in a network. In food webs, it refers to patterns of trophic interactions among taxa.

**Niche model:** A simple one-dimensional model of food web structure. *S* and *C* (Table 1) are used to specify the number of trophic species and links in a model web. Each species *i* is assigned a niche value *n_i_* drawn randomly and uniformly from the interval [0,1], and it consumes all species within a feeding range *r_i_* that is a segment of the interval, which is placed on the interval such that its center *c_i_* is equal to or lower than the niche value *n_i_* [13]. The niche model is notable for assuming a contiguous trophic niche for consumers.

**Probabilistic niche model:** A model that parameterizes the niche model directly to an empirical food web dataset [63],[64]. It produces an MLE of the fundamental niche model parameters (*n_i_*, *r_i_*, *c_i_*) for each species *i* in a given web. This allows computation of the probability of each link in an empirical web according to the model, and the overall expected fraction of links (ƒ_L_) predicted correctly (Table 1, Metric 22). It can be extended to more than one dimension.

**Scale dependence:** The empirically well-corroborated hypothesis that most food web structure metrics (Table 1, Metrics 6–22) and properties such as degree distribution change in systematic and predictable ways with the diversity (*S*) and/or complexity (*L/S*, *C*) of a food web (Table 1, Metrics 1–5). This scale dependence is built into models such as the MaxEnt and niche models through their use of *S* and *C* as the fundamental parameters. In addition, the fit of models to observed food webs also displays scale dependence, tending to decrease with increasing diversity or complexity.

**Specialist:** A consumer taxon that has very few possible resource taxa. In its strongest sense it refers to species that have specialized feeding on one other species.

**Robustness:** The proportion of primary extinctions that leads to a particular proportion of total extinctions, equal to primary plus secondary extinctions [17],[61]. A consumer species goes secondarily extinct if it loses all of its resource species. When assessed just based on food web network structure, robustness may be referred to more specifically as structural robustness.

**Trophic species:** Groups of taxa within a food web that share the same set of consumers and resources [65]. A trophic species web is generated from an original species web (i.e., the original dataset) by aggregating such taxa into single nodes. Most comparative food web structure studies focus on trophic species webs to reduce bias due to uneven resolution of taxa within and across food web datasets and to focus analysis and modeling on functionally distinct taxa.

**Vulnerability:** How many consumer taxa a resource taxon has.

Consistent with these types of expectations, prior studies of the network structure of food webs that include parasites have suggested that adding parasites alters food web structure [41]–[49]. This type of thinking is rapidly becoming conventional wisdom, as evidenced by a statement in a 2013 paper in *Trends in Ecology and Evolution* that “recent advances have shown that native parasites dramatically alter food web structure” [50]. However, there are two problems with this assertion. First, prior studies of parasites in food webs do not distinguish between changes in diversity and complexity and changes to network structure (Box 1). In food web studies, measures of diversity, such as species richness (*S*), and of complexity, such as link density (links per species, *L/S*) and connectance (the proportion of possible links actually observed, *C*), provide simple ways to characterize the numbers of nodes and links in those networks (Table 1, Metrics 1–4). However, in the general [51] and ecological [6] network literature, network structure refers to patterns of how links are distributed among nodes. As noted in a recent perspective in *Science*, “Network approaches to ecological research emphasize the pattern of interactions among species (the way links are arranged within the network)” [6]. While adding parasites, or any species, to food webs necessarily increases the numbers of species and links and can alter link density and connectance [45], such changes to diversity and complexity should not be characterized as changes in food web structure. Second, while adding parasites and their links generally does alter network structure properties, as noted by prior studies for a few metrics [41]–[49], there is usually an assumption that such changes result from unique aspects of parasite biology. However, those studies did not account for generic structural effects of adding any type of species and their links to a food web. One of the key insights of the last dozen years of comparative food web research regards the scale dependence (Box 1) of food web structure, which refers to the empirically well-supported hypothesis that most aspects of network structure change systematically with changes in the diversity and complexity of food webs, regardless of the identity of the species in the webs [52]–[56].

Thus, the overall hypothesis we test is whether changes to network structure arising from the addition of parasites to food webs are attributable to the unique trophic roles that parasites play in food webs, or, alternatively, are generic effects of adding any type of species and links to webs. We conducted comparative analyses of the structure of seven highly resolved food webs that include detailed metazoan parasite data [42],[57]–[60]. The food webs are from coastal areas and include a variety of habitats including estuaries, salt marshes, tidal basins, and mudflats. We assessed many metrics of food web structure (Table 1, Metrics 6–22) as well as degree distributions (Box 1) and motifs (Box 1), most of which have not been evaluated previously for food webs with parasites. To our knowledge, this is the broadest set of food web structure properties yet evaluated in a single study. Together they provide a wide range of ways to understand network structure, from system-level properties to types of taxa present in the system to local structure to the occurrence of specific links.

We did not analyze robustness (Box 1) [17],[61], as it has been explored extensively for food webs with parasites elsewhere [32]–[34], including an analysis of the seven food webs studied here [35]. That literature includes the only other study known to us that sought to disentangle generic from unique effects of parasites on network structure, by analyzing “whether the reduction in food web robustness after the inclusion of parasitism is due to factors associated with the characteristics of parasites, or simply an inevitable artefact of the addition of new nodes and links to an existing network” [34]. By comparing models with similar species richness (*S*) and connectance (*C*), that study showed that only those models that incorporated parasite life-cycle constraints resulted in substantial reductions in robustness as well as higher vulnerability of parasites to random species loss. Thus, the general finding of reduced robustness of food webs with parasites to species loss [32]–[35] was attributed to the complex life cycles of many parasites, rather than to generic changes in *S* and *C* [17],[54].

We also used a model-based strategy to assess whether changes in food web properties due to the addition of parasites are attributable either to their unique trophic roles or to generic effects of adding any species. The MaxEnt model for degree distributions [62], the niche model [12],[13], and the probabilistic niche model [63],[64] (see Box 1 for brief definitions of the three models) incorporate scale dependence. In particular, the MaxEnt and niche models use *S* and *C* as input parameters, while the probabilistic niche model matches *S* and *C* of empirical webs. The scale dependence of structure implicit in those models has been corroborated by analyses that show that these and related models generate networks with structure similar to that observed in empirical food webs [13]–[16],[62],[64]. The current study uses these models as a normalization tool—they provide a way to meaningfully compare the structural properties of empirical webs with different numbers of species and links, and they have been critical in identifying generalities in food web structure across space and time [10],[11],[54],[55]. In addition, these models display a fit to empirical data that is scale dependent, with decreasing model fit associated with food webs that have greater diversity and complexity. This second form of scale dependence of food web structure provides another way to assess whether parasites have generic or unique impacts on structure.

**Table 1:** Food web metrics.

Metric Number	Metric	Name	Definition
1	*S*	Species richness	Number of taxa (nodes) in a food web.
2	*L*	Trophic links	Number of feeding interactions (links or edges) between taxa in a food web. Trophic links are directional, such that “A feeds on B” is a separate link from “B feeds on A.”
3	*L/S*	Link density	Mean number of links per species.
4	*C*	Connectance	Proportion of possible trophic links that are realized. The most conventional algorithm is “directed connectance,” *C* = *L/S* ^2^, where *S* ^2^ is the number of possible links among *S* taxa, and *L* is the observed number of links [70].
5	*C*_adj_	Adjusted connectance	An alternate connectance measure, *C*_adj_ = *L/*(*F•S*), where *F* is the number of free-living species, used to measure connectance in food webs when excluding links from free-living to parasite species [45].
6	Top	Top taxa	Fraction of taxa that lack consumers.
7	Int	Intermediate taxa	Fraction of taxa that have both consumers and resources.
8	Bas	Basal taxa	Fraction of taxa that lack resource taxa.
9	Herb	Herbivores	Fraction of taxa that feed only on basal taxa. This includes detritivores, taxa that feed on detritus (non-living organic matter).
10	Omn	Omnivores	Fraction of taxa that feed on resource taxa that occur on more than one trophic level.
11	Can	Cannibals	Fraction of taxa that feed on individuals from the same taxon.
12	Loop	Species in loops	Fraction of taxa that occur in loops, excluding cannibals, e.g., when A eats B, B eats C, and C eats A, all three taxa occur in a loop.
13	LinkSD	Link number standard deviation	Standard deviation of the number of links per species.
14	GenSD	Generality standard deviation	Standard deviation of the number of resources per species.
15	VulSD	Vulnerability standard deviation	Standard deviation of the number of consumers per species.
16	TL	Trophic level	A measure of how many steps energy must take to get from an energy source to a focal taxon. Basal taxa are assigned TL = 1, obligate herbivores thus have TL = 2, and higher level consumers have TL averaged across the multiple food chains connecting them to basal taxa. The algorithm used here is “short-weighted trophic level,” the average of a consumer's shortest trophic level (1+shortest chain to a basal taxon) and its prey-averaged trophic level (1+the mean TL of all of its resources) [94].
17	MaxSim	Mean maximum similarity	The mean of all species' largest similarity index, which is calculated as the number of consumers and resources shared in common divided by the pair's total number of consumers and resources [13].
18	Path	Mean shortest path length	Mean of the shortest chain of feeding links (regardless of link direction) connecting each pair of taxa in a food web [8],[9]. A simple measure of how quickly effects can spread throughout a food web.
19	Clus	Clustering coefficient	Average fraction of pairs of species one link away from a particular species also linked to each other [8].
20	*ƒ*_G_	Degree distribution goodness of fit	Goodness of fit of a degree distribution, where ƒ_G_≤0.95 indicates that an empirical degree distribution is not significantly different from the model distribution at the 95% confidence interval [62].
21	*W*_95_	Degree distribution relative width	Relative width of a degree distribution, where −1≤*W*_95_≤1 indicates that an empirical distribution is neither significantly narrower (*W*_95_<−1) nor significantly broader (*W*_95_>1) than the distribution predicted by a model at the 95% confidence interval [62].
22	*f*_L_	Fraction of links	Fraction of specific links in an empirical food web predicted correctly by a model [63],[64].

To summarize, our study improves on prior studies in the following ways: it distinguishes changes in diversity and complexity from changes in network structure; it accounts for the generic effects of the addition of species and links on food web structure; it examines a wide range of local to system-level structural properties; it uses trophic species aggregation (Box 1) [65], which is a necessary step for model-based comparative analysis [10]–[16]; it considers the role of concomitant links (Box 1), the numerous trophic links that occur when a predator concurrently eats parasites infecting its prey [38],[47],[66]; and it analyzes seven highly resolved webs, compared to the one to five webs of previous studies, some of which lacked high resolution and/or comprehensiveness. Our results underpin a more comprehensive assessment than previously undertaken of whether adding parasites alters food web structure in unique ways and whether parasites play similar or different roles compared to other consumers and resources in ecological networks. Teasing apart the generic effects of increased diversity and complexity on observed food web structure from the specific effects of the unique topological roles of parasites, or other types of organisms not considered here, is an important and necessary step for developing a fundamental understanding of ecological networks that includes a more detailed accounting of the full diversity of ecosystems.

## Results

### Diversity and Complexity

We analyzed three versions of each web, one without parasites, one with parasites but no concomitant links (Box 1), and one with parasites and concomitant links. Each original species web version was aggregated into a trophic species web (Box 1), used as the basis for comparative network structure analyses. Species richness (*S*; Table 1, Metric 1) of the seven trophic species webs without parasites ranged from 56 to 117 (Table 2). The number of trophic links (*L*; Table 1, Metric 2) in the webs ranged from 358 to 1,085 (Table 2). Adding parasites increased *S* 1.2 to 1.9 times (range of 109 to 185) and *L* 1.4 to 3.4 times (range of 576 to 2,838), while adding concomitant links increased *L* 1.8 to 5.7 times (range of 1,252 to 4,671). *S* was reduced by seven to 33% and *L* by four to 51% in trophic species webs compared to original species webs (Table S1). The majority of the metazoan parasites (72% to 100%) in the original species webs have complex life cycles, where the parasites use two or more sequential hosts [27]. Those trophic shifts are often accompanied by an abrupt ontogenetic change in parasite morphology [67]. The use of sequential hosts by many of the metazoan parasites in these webs contrasts with the high degree of trophic specialization (i.e., only one host) reported for parasitoids in other ecological networks [68],[69]. In addition, the current webs have a large number of trematode parasites that tend to have relatively low specificity for the final host.

**Table 2:** Basic properties of trophic species food webs.

Food Web–Type	*S*	*L*	*L/S*	*C*	*C*_adj_	*S*_Free_	*S*_Par_	*S*_Bas_
Fals–Free	80	527	6.59	0.082	—	1.00	0.00	0.11
Fals–Par	141	1,792	12.71	0.090	0.138	0.65	0.35	0.06
Fals–ParCon	142	3,006	21.17	0.149	—	0.65	0.35	0.06
Carp–Free	91	761	8.36	0.092	—	1.00	0.00	0.10
Carp–Par	154	1,982	12.87	0.084	0.131	0.64	0.36	0.06
Carp–ParCon	154	3,350	21.75	0.141	—	0.64	0.36	0.06
Punt–Free	106	1,085	10.24	0.097	—	1.00	0.00	0.08
Punt–Par	185	2,838	15.34	0.083	0.131	0.63	0.37	0.05
Punt–ParCon	185	4,671	25.25	0.136	—	0.63	0.37	0.05
Flens–Free	56	358	6.39	0.114	—	1.00	0.00	0.11
Flens–Par	109	846	7.76	0.071	0.114	0.62	0.38	0.06
Flens–ParCon	109	1,252	11.49	0.105	—	0.62	0.38	0.06
Otag–Free	94	751	7.99	0.085	—	1.00	0.00	0.03
Otag–Par	117	1,054	9.01	0.077	0.090	0.85	0.15	0.03
Otag–ParCon	118	1,354	11.47	0.097	—	0.85	0.15	0.03
Sylt–Free	117	993	8.49	0.073	—	1.00	0.00	0.05
Sylt–Par	147	1,708	11.62	0.079	0.098	0.80	0.20	0.04
Sylt–ParCon	149	2,680	17.99	0.121	—	0.79	0.21	0.04
Ythan–Free	81	394	4.86	0.060	—	1.00	0.00	0.05
Ythan–Par	122	576	4.72	0.039	0.056	0.69	0.31	0.03
Ythan–ParCon	122	1,284	10.52	0.086	—	0.69	0.31	0.03

Fals, Carp, Punt, Flens, Otag, Sylt, and Ythan refer to the food webs for Bahia Falsa, Carpinteria Salt Marsh, Estero de Punta Banda, Flensburg Fjord, Otago Harbor, Sylt Tidal Basin, and Ythan Estuary, respectively. “Free” refers to webs with free-living species only; “Par” refers to webs with parasites but not concomitant links; “ParCon” refers to webs with parasites and concomitant links. *S*, *L*, *L/*S, C, and *C*_adj_ are defined in Table 1 (Metrics 1–5). *S*_Free_, *S*_Par_, and *S*_Bas_ refer to the fraction of taxa that are free-living, parasite, and basal, respectively.

Parasites comprised 15%–28% of taxa and were involved in 22%–74% of links, while free-living species were involved in 91%–100% of links in trophic species webs (Table S2), similar to original species webs (Table S3). Links can be divided into four categories based on the different possible relationships between free-living species (FL) and parasite species (Par): classic predation (FL-FL), classic parasitism (Par-FL), parasites consuming parasites (Par-Par), and predation of parasites (FL-Par) (Table S2). In trophic species webs with parasites, classic predation comprised 42%–78% of links, classic parasitism comprised 13%–38%, parasites consuming parasites comprised <10%, and predation of parasites comprised 0%–21%. Adding concomitant links decreased the shares of classic predation (26%–60%) and classic parasitism (1%–23%), barely altered parasites consuming parasites (<10%), and greatly increased predation of parasites (27%–52%). The number of classic predation links exceeded classic parasitism links except in the trophic species version of the Bahia Falsa web. The diversity of parasites of prey of free-living consumers resulted in predation-of-parasite links exceeding classic predation links in five of the seven webs with concomitant links.

**Figure 1:**
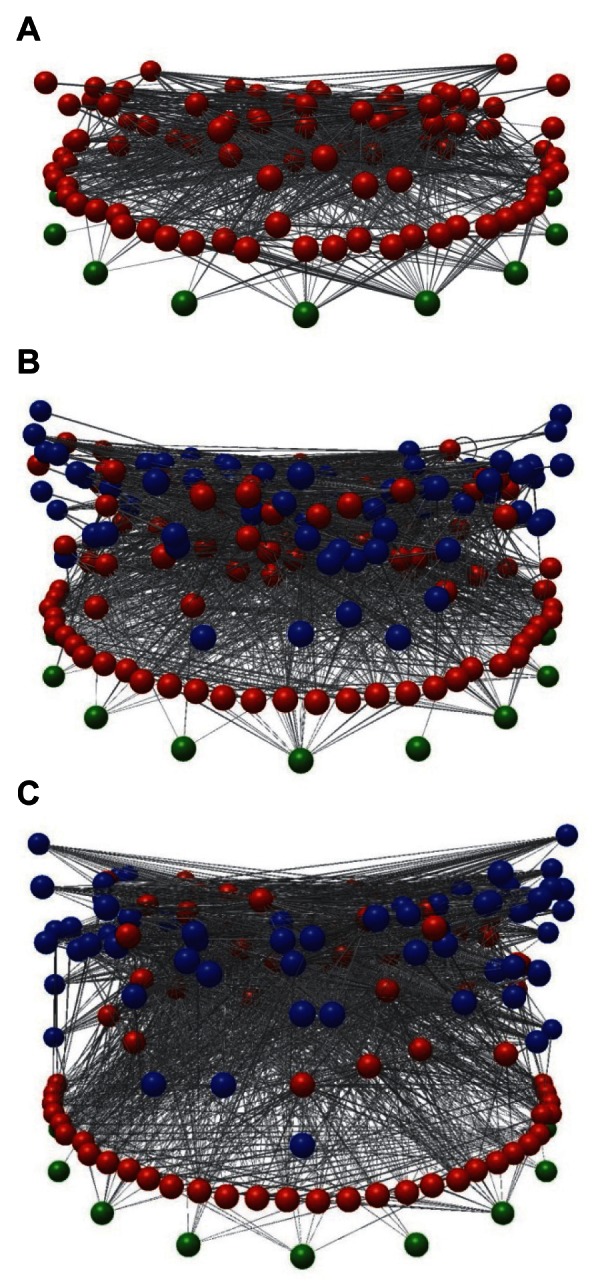
Images of three trophic species versions of the food web of Estero de Punta Banda. (A) Web with free-living species only. (B) Web with parasite species but not concomitant predation links. (C) Web with parasite species and concomitant links. Green indicates basal taxa, red indicates free-living taxa, and blue indicates parasites. The vertical axis corresponds to short-weighted trophic level [94]. The maximum trophic levels for a taxon in each web are 3.77 (A), 5.68 (B), and 7.16 (C). Images produced with Network3D software [95],[96], available by request from jdunne@santafe.edu.

The addition of parasites usually increased link density (*L/S*) and connectance (*C*) (Table 1, Metrics 3 and 4), and adding concomitant links resulted in further obligatory increases in *L/S* and *C* (Tables 2 and S1). The inclusion or exclusion of concomitant links changes the appropriate connectance measure to consider [45]. In webs that include concomitant links, the conventionally used “directed connectance” (*C* = *L/S* ^2^) is the appropriate measure, as it allows for the possibility of any link occurring between any two taxa [70]. In webs that exclude concomitant links, an “adjusted connectance” (*C*_adj_ = *L/*(*F•S*), where *F* is the number of free-living species) is the better measure (Table 1, Metric 5), as it accounts for the exclusion of links from free-living to parasite species, as discussed in detail elsewhere [45]. Example images of the Estero de Punta Banda trophic species food webs show how diversity and complexity increased as parasites and concomitant links were added to the food web (Figure 1).

### Degree Distributions

Degree distributions, the distribution of the number of links associated with each node, are a commonly studied feature of networks of all types [51]. For a given food web it is most useful to report separate resource and consumer distributions [10]. Resource distributions give the pattern of numbers of links each species has to its prey or host species, and thus describe the balance of trophic specialization and generality (Box 1) in an ecosystem. Consumer distributions give the pattern of numbers of links each species has to its predator species, and thus describe the balance of trophic vulnerability and invulnerability (Box 1) in an ecosystem. Most extant food webs studied thus far have cumulative degree distributions that map closely onto universal exponential-type scaling functions once data are normalized for link density (*L*/*S*) [8],[10]. The exponential shape indicates that the distribution of links in food webs is skewed across taxa [8],[10]—for example, most taxa are specialists (Box 1) that have one or a very few resources, while a few are generalists (Box 1) that have many resources [10]. The normalized cumulative degree distributions for resource (Figure S1) and consumer (Figure S2) links for the three versions of the seven webs studied here, with and without parasites, followed similar curves, with exponential-type shapes similar to those of previously studied webs [10]. The most variability appeared in the tails of consumer distributions, but the effect of adding parasites or concomitant links did not follow any particular pattern (Figure S2).

A more rigorous way to compare the shapes of these distributions, and to determine whether adding parasites alters the patterns of skewness of generality and vulnerability (Box 1) in food webs, is to assess to what degree they differ from the expectations of a null model, in this case, a MaxEnt model (Box 1). MaxEnt is a non-mechanistic statistical approach that predicts the most likely distribution of some property given known constraints on information about the system. It has been used successfully to predict various macroecological patterns [71]. When applied to food web degree distributions, MaxEnt produces distributions with an exponential shape similar to what has been observed previously in empirical food webs [62]. It provides a more ecologically realistic null scenario for evaluating and comparing food web degree distributions than models that distribute links randomly [72] and does not assume an exponential distribution like the niche model (Box 1) does [13].

**Figure 2:**
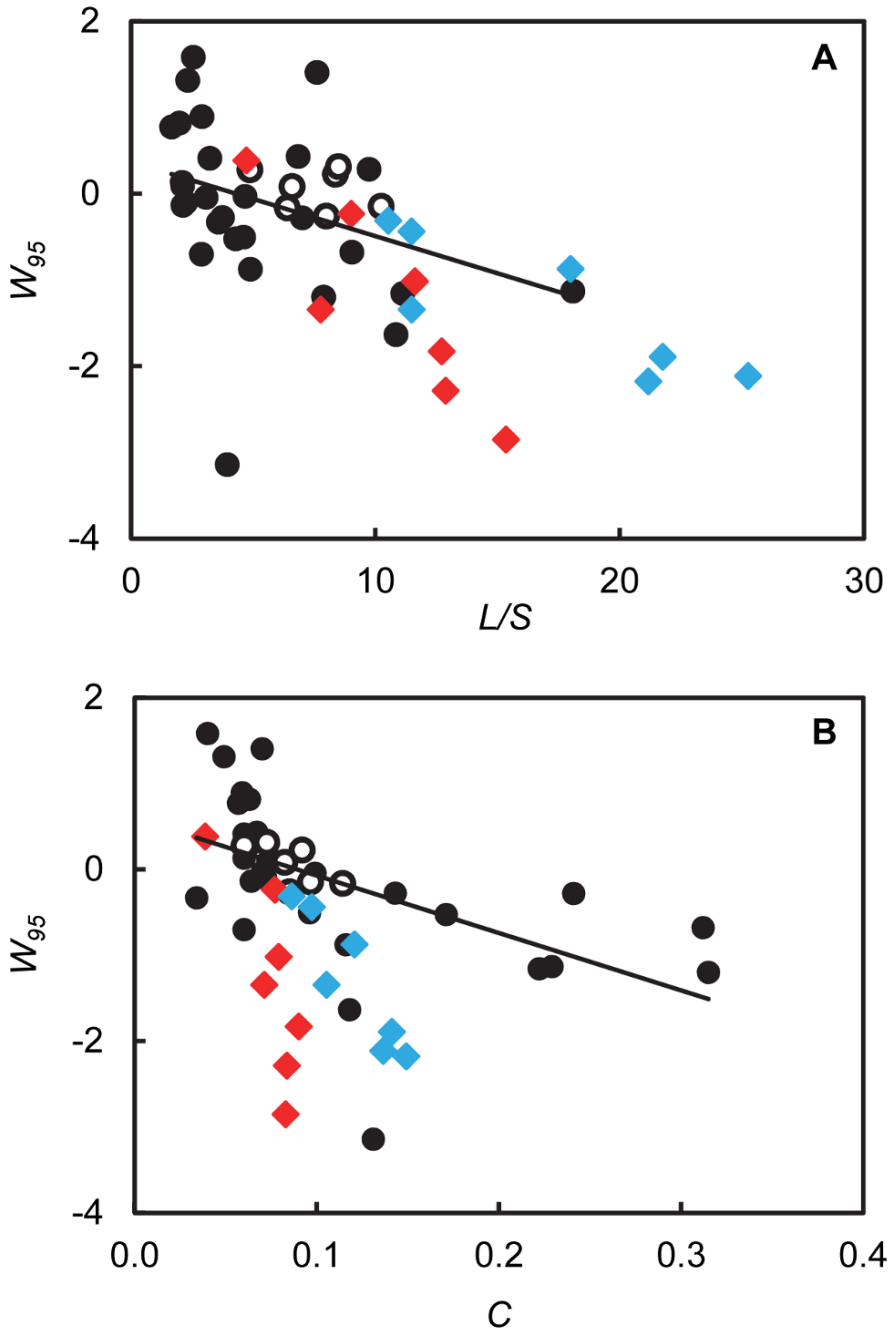
Scale dependence of MaxEnt model results. Relative width (*W*_95_) of the consumer distribution in relation to MaxEnt expectations, as a function of (A) *L/S* (links per species), and (B) *C* (directed connectance; *L/S* ^2^). Solid black circles show results for 28 previously studied free-living species webs (Table S5). Open black circles show results for the seven coastal free-living species webs analyzed in the current study. Red diamonds show results for the seven coastal webs with parasites but not concomitant links. Blue diamonds show results for the seven coastal webs with parasites and concomitant links. The black line shows the linear regression through the 35 free-living species webs.

Among the 21 current web versions, nine consumer distributions were significantly narrower, or less skewed, than MaxEnt expectations, in particular in webs with parasites, with or without concomitant links (Table S4). This means that in those nine food webs, the most vulnerable taxa (those consumed by the most species) had fewer consumers than expected compared to the most vulnerable taxa in the other 12 webs, whose consumer distributions did not differ from the MaxEnt expectation. Only one resource distribution, for the Flensburg Fjord web with both parasites and concomitant links, was significantly different (wider) than the MaxEnt expectation, meaning that its most generalist consumers fed on more species than expected compared to the other webs. Eight consumer and seven resource distributions were well fit by the MaxEnt model in terms of both the goodness of fit of the model *ƒ*_G_ and the expected width of the distribution *W*_95_ (Table 1, Metrics 20 and 21). Only two web versions (of the Ythan Estuary web) had both consumer and resource distributions well fit by the MaxEnt model. To evaluate whether the significantly narrower than expected consumer distributions for many webs with parasites were likely a result of the unique roles of parasites versus a result of scale dependence (Box 1) of network structure, we investigated a previously reported relationship between the width of the consumer distribution (*W*_95 Cons_) and *L/S* [62]. We combined the seven current webs without parasites with 28 prior food webs (Table S5; Methods S1) and found a significant decrease of *W*_95 Cons_ with *C* and a marginally significant decrease with *L/S* (Figure 2; Table 3). When results for webs with parasites were added, they were consistent with the observed scale dependence of *W*_95 Cons_ with *L/S* (Figure 2A), but fell below the scale dependence trend for *C* (Figure 2B). However, several previously studied webs without parasites also fell in a similar space below the trend line.

**Table 3:** Linear regressions for scale dependence of model results.

Metric	*W*_95 Cons_			|ME|			*f*_L_		
	*R* ^2^	*p*-Value	Slope	*R* ^2^	*p*-Value	Slope	*R* ^2^	*p*-Value	Slope
*S*	0.041	0.241	0.005	**0.541**	**0.001**	**0.009**	**0.532**	**<0.001**	**−0.003**
*L*	0.004	0.720	−0.0001	0.300	0.023	0.001	**0.266**	**0.002**	**−0.0001**
*L/S*	0.118	0.044	−0.086	0.054	0.370	0.025	0.081	0.097	−0.009
*C*	**0.290**	**0.001**	**−6.682**	0.160	0.112	−1.827	0.127	0.035	0.568

The *R* ^2^, *p*-values, and slopes for linear regressions of the dependent variables *W*_95 Cons_ (width of the consumer resource distribution in relation to MaxEnt expectations), |ME| (absolute value of the average niche ME), and *f*_L_ (fraction of links correctly predicted by a one-dimensional probabilistic niche model), as a function of the explanatory variables *S*, *L*, *L/S*, and *C* (Table 1, Metrics 1–4). Each regression includes the seven free-living species webs currently analyzed and 28 (*W*_95 Cons_, *f*_L_) or ten (|ME|) additional food webs (Table S5). Regressions that are significant at a Bonferroni-corrected (*n* = 4) *p*-value of 0.0125 are shown in bold.

### Network Structure Properties

**Figure 3:**
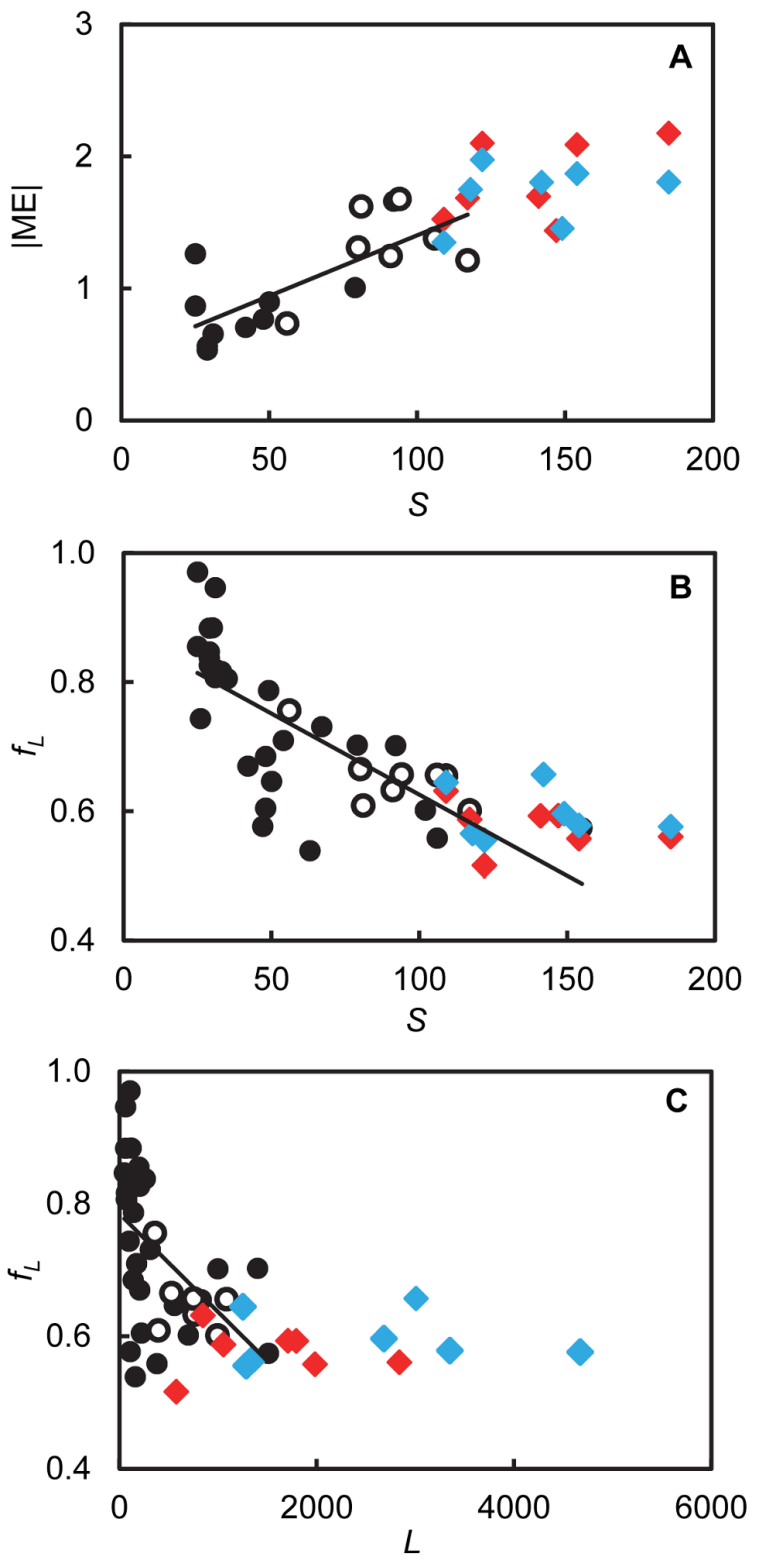
Scale dependence of niche and probabilistic niche model results. (A) Mean absolute niche ME (|ME|) for 14 properties as a function of *S*. (B) The fraction of observed links (ƒ_L_) predicted by the one-dimensional probabilistic niche model as a function of *S*. (C) The ƒ_L_ predicted by the one-dimensional probabilistic niche model as a function of *L*. Solid black circles show results for ten (A) or 28 (B) previously studied free-living species webs (Table S5). Open black circles show results for the seven intertidal free-living species webs analyzed in the current study. Red diamonds show results for the seven intertidal webs with parasites but not concomitant links. Blue diamonds show results for the seven intertidal webs with parasites and concomitant links. The black line shows the linear regression through the free-living species webs.

In terms of 14 commonly studied network structure properties that have well-documented ecological meaning and associated bodies of research (Table 1, Metrics 6–19), the niche model (Box 1) [13] fit the webs relatively poorly, especially when parasites were added. Model errors (MEs) for properties related to types of taxa (Table 1, Metrics 6–12) show that for one-third or more of the 21 webs the niche model significantly underestimated the fractions of taxa that are top species, that are herbivores, and that occur in loops, and significantly overestimated the fractions of basal taxa, omnivores, and cannibals (Table S6). For other web properties the niche model often significantly underestimated the variability in the number of links per species and the number of consumers per species, as well as mean trophic level (Table S7). It generally overestimated the mean maximum trophic similarity of pairs of species (Table S7). Across all 14 properties, webs without parasites had the most properties well fit by the niche model (mean = 8.14), compared to webs with parasites (mean = 4.86) and webs with parasites and concomitant links (mean = 6.14). However, the reduced fit of the niche model in webs with parasites compared to webs without parasites appears consistent with scale dependence of model fit. When the current seven web versions lacking parasites were combined with ten previously studied webs (Table S5; Methods S1), there was a significant increase in mean absolute ME with *S* and a marginally significant increase with *L* (Table 3; Figure 3A), consistent with prior results [12]. Niche model results for webs with parasites were consistent with the observed scale dependence of mean absolute niche ME with *S* for webs without parasites (Figure 3A). In other words, as species richness increases, the fit of the niche model decreases, and there is no evidence that webs with parasites deviate from this trend.

### Network Motifs

**Figure 4:**
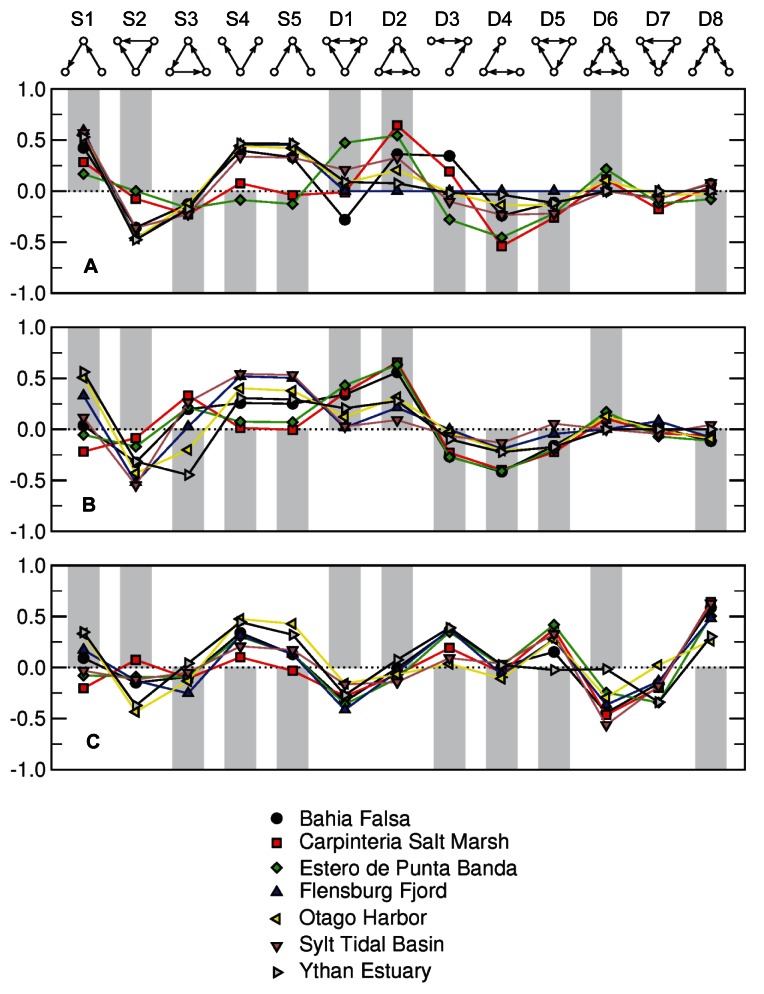
The representation of three-node motifs in three versions of each of the seven food webs. (A) Results for webs with free-living taxa only. (B) Results for webs with parasites but not concomitant links. (C) Results for webs with parasites and concomitant links. Motif labels and graphics are shown at the top of the figure, with arrowheads pointing from resources to consumers. The data points show the normalized profile overrepresentation (>0) or underrepresentation (<0) of each motif in the seven food webs. The grey bars show either predicted overrepresentation (>0) or underrepresentation (<0) of the individual motifs in niche model webs.

For three-node motif (Box 1) representation—the frequency with which every possible pattern (13 in total) of interactions among three species occurs in a web relative to its frequency in randomized webs—the seven food webs without parasites showed patterns similar to the typical pattern exhibited across most previously analyzed food webs and in the niche model (Figures 4A and S3A) [11]. The most notable differences were underrepresentation of omnivory (motif S2) and overrepresentation of exploitative and apparent competition (motifs S4 and S5). These deviations, however, were also observed in a few previously studied food webs [11]. Adding parasite links resulted in a similar overall pattern (Figure 4B). This result suggests that interactions involving parasites were distributed across motifs in a manner similar to that of interactions involving free-living species, as confirmed by the results of the compartmented randomization (Figure S3B). However, the addition of concomitant predator–parasite links substantially changed the motif pattern (Figure 4C). These changes were most pronounced in motifs D1 to D8 and indicate that bidirectional interactions made up of one parasite–host interaction and one concomitant link are distributed differently across motifs involving free-living species links and appear far more frequently in some motifs than in others. This observation was confirmed by marked differences between patterns of motif representation when webs with concomitant links were compared across the standard and compartmented randomizations (Figures 4C and S3C). In the compartmented randomization, the addition of concomitant links also changed the over- and under-representation of motifs S1 to S5 to a pattern inconsistent with all empirical webs previously studied [11], as well as the currently studied webs without parasites and webs with parasites but not concomitant links. These results suggest that patterns of prey selection in food webs were altered by the addition of parasites and concomitant links from predators to the parasites of their prey [11], as a result of the trophic intimacy of parasites with their hosts.

### Link Probabilities and Trophic Niche Structure

A recently proposed probabilistic niche model (Box 1) uses maximum likelihood methods to parameterize the niche model directly against food web data [63],[64]. It returns parameter estimates for each species in a web, and relaxes niche model assumptions about parameter distributions and hierarchical ordering of taxa. It also provides a probability of each link occurring, which can be compared to the actual links observed. A one-dimensional probabilistic niche model correctly predicted 0.601 to 0.756 (mean ƒ_L_ = 0.654) of links for webs without parasites, 0.516 to 0.631 (mean ƒ_L_ = 0.577) of links for webs with parasites but no concomitant links, and 0.555 to 0.657 (mean ƒ_L_ = 0.596) of links for webs with parasites and concomitant links (Table S8). In each of the seven empirical food webs, ƒ_L_ was ∼10%–20% greater for webs without parasites than for webs with parasites, indicating a significantly lower ƒ_L_ in webs with parasites (binomial test, seven of seven food webs, *p* = 0.0156). In most cases, ƒ_L_ was similar for webs with parasites with or without concomitant links. A two-dimensional probabilistic niche model resulted in greater ƒ_L_ for all 21 web versions, ranging from 0.624 to 0.927, with means of 0.801, 0.737, and 0.758 for webs without parasites, with parasites, and with parasites and concomitant links, respectively. Decreases in Akaike Information Criterion values indicated that the two-dimensional model performed better than the one-dimensional model for all 21 web versions (Table S8). However, the decrease in the fraction of links correctly predicted by the probabilistic niche model from webs without parasites to webs with parasites appears consistent with scale dependence of model fit. When the current seven webs without parasites were added to 28 previously studied webs (Table S5; Methods S1), ƒ_L_ significantly decreased with both increasing numbers of species (*S*) and links (*L*) (Figure 3B and 3C; Table 3), consistent with prior results [64]. The results for the current webs with parasites with or without concomitant links were consistent with the observed decrease of ƒ_L_ with increasing *S* (Figure 3B). For webs with >1,500 links (i.e., most of the webs that include parasites), a minimum ƒ_L_ of ∼0.50 appeared to hold (Figure 3C). A possible lower bound on ƒ_L_ in relation to *L* was suggested in an earlier study [64].

**Figure 5:**
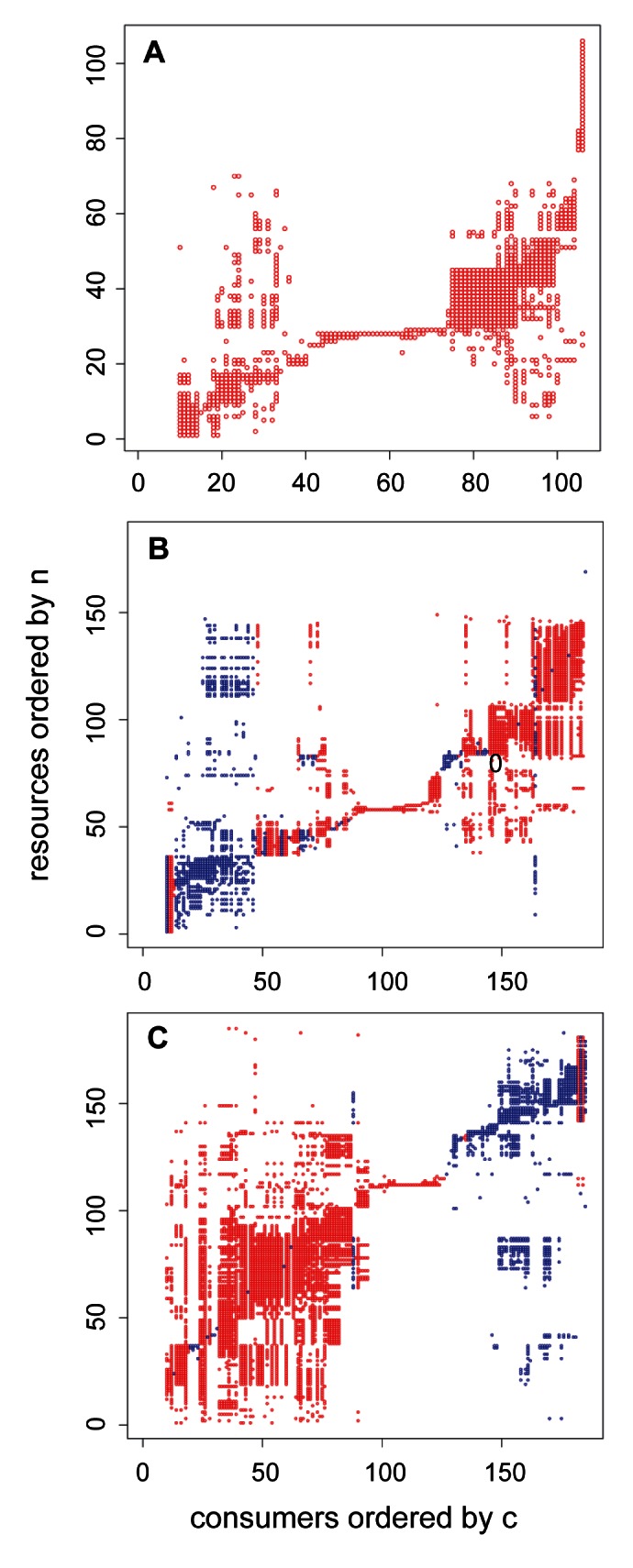
Visualization of trophic niches of species in Estero de Punta Banda food webs. MLE values for consumer niche position (*c*) are on the *x*-axis and for resource niche value (*n*) are on the *y*-axis. (A) Results for the web with free-living species only. (B) Results for the web with parasites but not concomitant links. (C) Results for the web with parasites and concomitant links. Red dots show the resource links for free-living consumers, and blue dots show the resource links for parasite consumers.

Using maximum likelihood estimates (MLEs) of niche model parameters, we ordered consumers by the position of their feeding range (*c_i_*) along the *x*-axis in Figure 5, with their resources ordered by their niche value (*n_i_*) along the *y*-axis, and then marked documented links at the intersection of consumers and resources. This provides visualization of whether the resources of generalists tend to be dispersed along the niche axis or are concentrated with a near-contiguous core (referred to hereafter as “trophic niche structure”), and whether parasite feeding ranges tend to clump or disperse along the niche axis (Figure 5). The trophic niche structure of generalists in the web without parasites showed that their resources' most likely niche values tended to arrange in a nearly contiguous core interval of niche space (Figure 5A), with gaps (i.e., discontinuities in a column of links) occurring more frequently towards the edges of the consumer's trophic niche, consistent with previously studied webs [64]. When parasites were added, the most likely feeding range positions of most parasites tended to group together (Figure 5B). The parasites with multiple hosts also displayed a core trophic niche structure, but compared to those of generalist free-living consumers, parasites' links to resources spread across a larger interval of niche space, there were more gaps in their trophic niches, and in some cases there appeared to be secondary trophic niches separated from the main trophic niche. When concomitant links were added (Figure 5C), the parasites with multiple hosts displayed similar patterns, and the breadth of trophic niches of generalist free-living species expanded greatly but still appeared to have a single nearly contiguous core. All seven webs displayed qualitatively similar patterns (Figures 5 and S4, S5, S6).

## Discussion

Prior claims that parasites affect food web structure differently from free-living consumers either focused on changes to diversity and complexity when parasites were added, or did not control for the effects of increases in diversity and complexity on network structure properties. Our study clarifies the distinction between changes in food web diversity and complexity and changes in food web structure, which consists of the patterns of how feeding links are distributed among species [6]. We assessed both aspects of change in food webs when parasites were added, as discussed separately below.

Our most novel and important findings concern network structure, and whether observed changes in structure result from increases in diversity and complexity when parasites are included, or instead are attributable to the unique roles that parasites play in food webs. In particular we show how the addition of parasites to food webs changes most aspects of local to system-level structure in ways primarily attributable to the generic effects of increases in diversity and complexity, regardless of the identity or type of species and links being added. However, our analyses identify two ways in which parasites do appear to play unique topological roles in food webs. First, in their roles as resources, they have close physical intimacy with their hosts, and thus are concomitant resources for the same predators. Second, in their roles as consumers, they can have complex life cycles and inverse consumer–resource body-size ratios, different from many free-living consumers. These unique roles of parasites in food webs resulted in alteration of the frequency of motifs in the case of their roles as resources, and differences in the breadth and contiguity of trophic niches between parasites and free-living species in the case of their roles as consumers.

These findings can be added to one other rigorously identified unique effect of parasites—their impact on robustness. Several studies have reported that the addition of parasites reduces food web robustness to species loss [32]–[35]. One study found that reductions in robustness associated with parasite additions are not explained by species richness and connectance, known to affect robustness [17],[61], but are explained by parasites' complex life cycles [34]. That study and the current study highlight the importance of disentangling the generic structural effects of adding species and links to food webs from the unique effects attributable to the characteristics of parasites, or any other type of species being investigated.

### Diversity and Complexity

Our analyses corroborate previous findings for how parasites alter diversity and complexity of food webs [45]. As occurs with the addition of any species to food webs, adding parasites to the trophic networks studied here increased the number of species (*S*) and links (*L*), and also usually increased link density (*L/S*). Increases in links and link density were especially dramatic with the inclusion of concomitant links, the numerous links from predators to the parasites of their prey. Adding parasites also increased connectance (*C*) in most of the food webs analyzed here, especially when concomitant links were included or when connectance was adjusted to account for the non-inclusion of those links [45]. However, our study offers clarification of a prior finding that parasites “dominate” food web links, based on a comparison of classic parasitism links to classic predation links in an earlier version of the Carpinteria Salt Marsh web [45]. For the current seven webs, classic predation links outnumbered classic parasitism links in most cases, including in the Carpinteria Salt Marsh web. Overall, parasites were sometimes involved in >50% of food web links, particularly as prey when concomitant links were included, but free-living taxa were always involved with >90% of links because the vast majority of parasite links included free-living species. Thus, strictly speaking (and by necessity), free-living species are involved in more food web links than are parasites. However, parasites are involved in substantial fractions of food web links, and if excluded, datasets would often account for less than 50% of the links in a given food web.

It is important to note that any particular observation of the proportions of types of taxa and links, and thus the relative “dominance” of particular types of taxa or links, can be strongly influenced by the levels of taxonomic and trophic resolution [70] and sampling intensity [68],[73],[74] of the ecological networks in question. For example, in the current seven food web datasets, free-living bacteria and protozoa are either absent or highly aggregated. However, parasitic bacteriophages and protozoa are also absent. When we consider that worldwide, ∼60,000 vertebrate species may host ∼300,000 parasite species [21], undersampling likely leads to greater underestimates of parasites and their links than of free-living species.

### Network Structure: Generic Changes

Prior studies have shown that variability in the raw values and distributions of network structure properties, as observed for food webs with and without parasites, often masks generalities in ecological network structure. Such generalities emerge only after appropriate normalization for diversity and complexity [8],[10],[53]. The MaxEnt, niche, and probabilistic niche models (Box 1) are used in this study as tools that provide normalizations that allow comparison of the structure of webs with different numbers of species and links. These models have previously performed well, revealing generalities in the structure of food webs [10]–[13],[54],[62],[64]. In this study, the models generally did a worse job describing the structure of food webs with parasites than food webs without parasites. This would seem to corroborate prior assertions that adding parasites alters food web structure in unique ways [41]–[48].

However, the webs with parasites in this study have species richness values of 109 to 185, greater than that of most webs without parasites previously studied. Each of the models used to evaluate network structure in our study has known scale dependence with diversity and complexity, such that the fit of the models decreases in relation to *S*, *L*, *L/S*, or *C* of the empirical web being analyzed [12],[62],[64]. When the current seven webs without parasites are compared to prior webs that lack parasites, significant scale dependencies of model fit are corroborated and extended: the width of the consumer distribution narrows with *C* and *L/S*; the absolute mean niche ME increases with *S* and *L*; and the fraction of links correctly predicted by the probabilistic niche model decreases with *S* and *L* (Table 3). The network structure of webs with parasites is in most cases consistent with these scale dependencies observed in webs without parasites (Figures 2 and 3). This suggests that apparent differences in several commonly studied aspects of network structure for webs with and without parasites are not attributable to special topological roles that parasites might play in food webs. Instead, they appear to result from generic changes in network structure due to the increasing diversity and complexity of food webs when parasites are added.

Specifically, we found that changes in consumer and resource distributions, 14 commonly studied food web metrics, food web motifs (when concomitant links are excluded), and link probabilities are consistent with generic changes in food web structure associated with changes in diversity and complexity, regardless of species identity. Also, in prior work, relative nestedness, a measure of network structure not considered in the current analysis, was found to change very little with inclusion of parasites and classic parasitism links [45]–[47], but it increased greatly with the further inclusion of concomitant links in the Carpinteria Salt Marsh web [45]. This change may be attributable to a positive relationship of nestedness with connectance [74],[75], which increases with the addition of concomitant links. This should be investigated more explicitly with regard to scale dependence in future research.

Our findings suggest that many aspects of previously identified generalities in food web structure across habitats and deep time [10],[11],[54],[55] likely extend from free-living species food webs to those that include parasite species. This is consistent with macroecological patterns showing that parasites and free-living species play by similar rules when it comes to the relationship between body size, abundance, and trophic level [23], in addition to similarities observed in other aspects of the metabolic theory of ecology [24]. Our analyses do highlight some patterns that need clarification with more data in the future. Specifically, a possible lower bound on the fraction of links correctly predicted by the probabilistic niche model (ƒ_L_∼0.50) at ∼1,500 links, as suggested by webs with parasites, needs to be examined for other webs without parasites, but with high numbers of links. Also, the rate of decrease in the width of consumer distributions with increasing connectance needs to be clarified with additional data for webs with *C*>0.1. In general, because the scale dependencies based on webs without parasites reflect ranges of species richness and numbers of links lower than those for webs with parasites, additional data for more diverse webs without parasites, as well as highly resolved webs with parasites from other habitats, will allow more rigorous assessment of the scale dependence of model fit and whether webs with parasites are as consistent with those trends as initially indicated by this study.

This brings us to another important point—our analyses reveal limitations of current simple models of food web structure. The majority of webs used to evaluate network structure thus far generally have trophic species richness less than 100. The simple models used here and elsewhere appear to fit the structure of food webs with *S*<100 reasonably well, but, as we show, that fit decays systematically with increased diversity and/or complexity of the food web [12],[62],[64]. Our results suggest that the availability of more diverse, comprehensive, and highly resolved data requires development and testing of new network structure models, and may require a shift from low- to higher-dimension approaches.

### Network Structure: Unique Changes

Beyond generic scale-dependent effects of greater diversity and complexity on network structure and model fit when parasites are added, two of our analyses suggest that parasites play certain unique topological roles in these food webs. First, the addition of parasites with concomitant links resulted in large and consistent differences in motif representation compared to webs without parasites, webs with parasites but no concomitant links, and niche model webs, all of which had similar motif frequencies. This was especially the case for motifs that included at least one set of two-way (bidirectional) links between a pair of taxa. These results imply that, topologically, the roles of free-living species as prey are similar whether they are consumed only by free-living species or by parasites. However, the roles played by parasites as concomitant prey are substantially different from the roles played by free-living species as prey or hosts. This is attributable to the close physical intimacy of parasites with their hosts [26], which ensures that parasites are also eaten when their host is eaten, something that is generally not the case for classic predator–prey interactions. Thus, inclusion of concomitant links increases the amount of intraguild predation, predation that occurs between taxa that feed on the same prey species [76],[77]. However, it increases such predation only from predators to parasites, and not the reverse, and these patterns would be useful to quantify in future research.

Second, analysis of the most likely trophic niche structure of species reveals some differences between parasites and free-living species. While most generalist consumer species, whether free-living or parasite, tend to have a core, near-contiguous trophic niche with gaps occurring more frequently towards the edges of the range [63],[64], the trophic niches of parasites tend to be broader and have more gaps, and in some cases parasites display a smaller, secondary trophic niche. Also, the positions of the trophic niches of parasites tend to group together and are not dispersed throughout the niches of free-living species. A contiguous or near-contiguous trophic niche is a central assumption of the niche and related models [13]–[16], with near contiguity observed in empirical data [78]. The weakening of the near-contiguous trophic niche pattern for parasite species, including occasional secondary trophic niches, may result from the complex life cycles of many parasites [42]. Parasites can have multiple hosts that diverge from each other in a variety of ways such as body size and phylogeny, factors that are thought to be important for structuring food webs [15],[79],[80]. As an example, trematodes are a common parasite group in most of the webs we examined. They use mollusks as first intermediate hosts, fish and invertebrates as second intermediate hosts, and fishes and birds as final hosts [57]–[60].

The inability of the one-dimensional probabilistic niche model to assign a strong contiguous trophic niche to many parasites, and the fact that it tends to group parasites together, may also be related to body size. While free-living consumers are usually larger than their resources by one or more orders of magnitude [81], parasites are smaller than their resources by similar orders of magnitude [82], which may result in parasites' feeding being less restricted to contiguous ranges of body sizes. The single niche dimension embodies the concept of a hierarchical species ordering. Body size is a favored hypothesis for how taxa may be ordered [79], but inclusion of parasites will disrupt any single-dimensional body-size-based ordering in a food web [23],[42]. Even for webs without parasites, the importance of body size can vary substantially across webs [83],[84], and hierarchical ordering itself may often not apply [64].

Increases in intraguild predation and the inclusion of species that lack strongly contiguous, one-dimensional trophic niches should tend to drive food web structure away from niche model expectations. However, our findings suggest that such shifts may be dominated and masked by concurrent scale-dependent shifts in network structure. Future research could address how much additional intraguild predation as well as deviations from niche contiguity, both of which appear to be associated with parasites in food webs, are required to noticeably shift network structure patterns such as link distributions and structural metrics away from empirical and model expectations. Also, future work should focus on more quantitative assessment of patterns and relationships of probabilistic niche model parameter estimates. Such research could quantify differences in the contiguity of the trophic niches of parasites versus free-living predators in one and two dimensions, as well as differences in the contiguity of the trophic niches of free-living consumers with and without inclusion of concomitant links. These analyses would be one way to test the hypothesis presented here, that parasites tend to have more complex trophic niches than free-living taxa.

### Implications for Future Research

Our work provides a framework for evaluating future claims that adding any particular type of species changes food web structure in unique ways. For example, protozoa, endosymbionts, bacteria, and viruses have yet to be adequately represented in food webs, and, like parasites, are small, can be cryptic, and can be subject to concomitant predation. Terrestrial insects and their interactions are thus far very poorly resolved in food webs, and primary producers are often aggregated. The impact of fixing any of these or other biases on ecological network structure has to be assessed relative to generic impacts of altering the diversity and complexity of food webs [29],[54],[55]. In addition, the impact of parasites on the network structure of terrestrial systems may be different from that observed in the coastal aquatic systems analyzed here if terrestrial parasites tend to play significantly different kinds of roles as resources and consumers in those systems compared to estuary or marine-based parasites.

The current findings also have important implications for modeling. The inverse niche model was recently proposed for food webs with parasites [85]. This model assigns links between parasites and hosts by inverting two niche model rules [13]. First, the parasite's niche value (*n_i_*) and feeding range (*r_i_*) are assigned as usual, but the position of the feeding range (*c_i_*) is higher, rather than lower, than the parasite's *n_i_*, resulting in a reverse hierarchy for parasites. Second, the size of parasites' *r_i_* decreases, and thus specialization increases, as parasites' *n_i_* increases. The niche model's assumption of trophic niche contiguity still holds—parasites feed on all taxa in their feeding range. Free-living species follow the usual niche model rules. While this model, which treats parasites differently from free-living species, was not compared directly to a niche model that does not distinguish between parasites and non-parasites (i.e., the way the niche model was implemented for the current analyses), it did fit data for Carpinteria Salt Marsh better than various null models. The current results suggest that if parasites are treated differently in models, the assumption of contiguous parasite feeding niches should be altered to account for greater breadth, more gaps, and the occasional presence of secondary niches. Alternatively, focusing on life stages with distinct diets as nodes in food webs may resolve this issue. Also, the inverse niche model excluded parasite–parasite links and any consumption of parasites by free-living species. Food web data should document, and associated models should allow for, the potential occurrence of links between any two taxa, which then sets directed connectance (*C* = *L/S* ^2^) as the appropriate connectedness measure. In the webs studied here, there are instances of all types of interactions, including more uncommon links such as free-living species feeding on free-swimming parasitic stages.

Producing an empirically well-supported model of the network structure of food webs with parasites and all types of links will also be important for dynamical modeling of parasites in food webs. Obvious questions are how parasites augment or inhibit the dynamical persistence and coexistence of species, and how parasites alter the likelihood of secondary extinctions given bottom-up, top-down, and indirect effects. For example, one approach to modeling food web dynamics starts by generating network structure with the niche model or a similar model and then implements nonlinear bioenergetic equations constrained by metabolic scaling and allometric relationships to model the biomass dynamics through time of each species in that network [86]–[89]. This approach needs to change when parasites are included to reflect the topological differences noted in this study, without violating the strong scale dependence of many features of food web structure.

Other differences between parasite–host, predator–prey, and predator–parasite relationships will need to be integrated in future models, such as differences in consumer–resource body-size ratios, the role of host as both food and habitat for parasites, the role of concomitant links, the complex life cycles of parasites, and potential differences in biomass flow between predators and prey and parasites and hosts. Key emerging aspects of global change research include understanding how interactions among organisms mediate ecological function at multiple scales [5],[7], as well as understanding the dynamic relevance of the structural roles of species [90]. Given the diversity of parasites in every ecosystem and at every trophic level, future food web models used in global change studies need to better encompass the topology and dynamics of complex interactions among parasites and free-living species, while also taking account of well-supported scale dependencies of network structure and model fit.

## Materials and Methods

### Data

We analyzed seven highly resolved coastal marine or estuarine food webs with detailed metazoan parasite data. Three North American Pacific coast webs were recently compiled by one research group [57]: Carpinteria Salt Marsh in California, US (an earlier version was published in [45]); Estero de Punta Banda in Baja California, Mexico; and Bahia Falsa in Bahia San Quintín, Baja California, Mexico. Three additional coastal webs in Europe and New Zealand were recently compiled by a second research group: Flensburg Fjord on the Baltic Sea between Germany and Denmark [58]; Sylt Tidal Basin on the North Sea between Germany and Denmark [59]; and Otago Harbor in Dunedin, New Zealand [60]. A seventh food web published in 1996 for the Ythan Estuary on the North Sea near Aberdeen, Scotland [42], was also used, as it has a resolution of free-living taxa and metazoan parasites comparable to that of the other six webs. This set of seven webs with parasites has been analyzed in one other paper focused on the effects of including parasites in food webs on food web robustness [35]. We excluded from analysis two freshwater webs with parasites [46],[47] because they have lower diversity and resolution.

In general, the compilation of data for the seven webs used in this analysis made use of consistent methodologies for identifying links [91]. Individuals of free-living species sampled in each habitat were dissected to identify metazoan parasites. This approach was combined with a strategy that emphasized searching for more individuals of rare free-living species to reduce the bias towards underrepresentation of parasites of uncommon hosts. These directly sampled data were augmented with literature-based data for the particular sites or nearby sites, as well as with inferences based on current understanding of host and parasite biology. Another bias that leads to underestimation of parasite diversity is the non-identification of certain classes of parasites altogether. For example, in the seven webs analyzed here, bacteriophages and protozoans were either not identified or were under-identified. Both of these biases, underreporting rare taxa and failing to resolve or include whole groups of cryptic or small taxa (e.g., microbes), are a problem for both parasite and free-living taxa, but likely result in greater underestimation of parasite diversity, given the fact that most host taxa have more than one parasite species.

The original seven datasets [42],[57]–[60] included ontogenetic life stages of parasite species with complex life cycles as separate food web nodes. However, for our analysis we aggregated parasite life stages and their feeding links into a single parasite node and set of links [92]. While species-level analysis masks temporally distinct resource use by many parasite taxa whose juvenile and mature forms have different diets, comparative studies of food web structure generally use the species as the lowest level of resolution, and ontogenetic diet data are not yet available for most free-living species, some of which also undergo ontogenetic and trophic life-stage shifts.

We analyzed data for three versions of each food web [92]: a free-living species web, a web with parasites but no concomitant links, and a web with parasites and concomitant links. Concomitant links were inferred by assuming predators eat all parasites of infected prey. All datasets except for Ythan Estuary also included some documentation of parasite–parasite links and targeted (non-concomitant) consumption of parasites by free-living species. We focused our analyses on the trophic species (Box 1) versions of the 21 webs.

### Analyses

For each web, we generated cumulative degree distributions (Box 1) across species for the number of links from predators (“consumer distribution”) and links to prey or hosts (“resource distribution”) per node, normalizing the link counts by *L/S* for each web [8],[10]. We tested the fit of a maximum information entropy MaxEnt model for food web degree distributions (Box 1) [62] to empirical food web link distributions. MaxEnt models generate the least biased probability distributions by maximizing the information entropy for a system after applying information-containing constraints. For food web degree distributions, *S* and *C* serve as such constraints, and we included an additional constraint, the number of basal species for resource distributions and the number of top species for consumer distributions [62]. We tested the fit of MaxEnt predictions by calculating goodness of fit, ƒ_G_, and relative width of the degree distribution, *W*_95_ (Table 1, Metrics 20 and 21). ƒ_G_≤0.95 indicates that the empirical web's link distribution does not differ significantly from the model distribution at the 95% confidence interval [62]. When −1≤*W*_95_≤1, the empirical distribution is neither significantly narrower (*W*_95_<−1) nor significantly broader (*W*_95_>1) than the distribution predicted by the model at the 95% confidence interval. A distribution is considered well fit by a model when both criteria are met: ƒ_G_≤0.95 and −1≤*W*_95_≤1.

We calculated link density (*L*/*S*) and directed connectance (*C* = *L/S* ^2^) for each web, as well as adjusted connectance (*C*_adj_ = *L/F•S*) (Table 1, Metrics 3–5) for webs with parasites but no concomitant links, to account for exclusion of such links in those web versions [45]. We calculated 14 network structure properties [12],[55] for each web (Table 1, Metrics 6–19): the fractions of top, intermediate, and basal species (Top, Int, Bas); the fractions of cannibals, herbivores, omnivores, and species in loops (Can, Herb, Omn, Loop); the standard deviations of normalized total links, generality, and vulnerability (LinkSD, GenSD, and VulSD); the mean short-weighted trophic level of all species (TL); the mean maximum trophic similarity of species (MaxSim); the mean shortest number of links between species pairs (Path); and the mean clustering coefficient (Clus). We generated 1,000 niche model webs with the same *S* and *C* as the 21 webs, and for each property for each web, calculated ME, the normalized difference between the model's median value and the empirical value [12]. ME>|1| indicates that the empirical property falls outside the most likely 95% of model values, with negative and positive MEs indicating model underestimation and overestimation of the empirical value, respectively.

We investigated over- and underrepresentation of the 13 unique motifs (Box 1) that can occur among three species [11]. Motifs S1 to S5 include only single-directional links between taxa pairs, while motifs D1 to D8 include bidirectional links (i.e., mutual predation) between at least one species pair. The frequency of a motif in an empirical food web was compared to the same in an ensemble of randomized webs, yielding a *z*-score for each motif *i* that measures the degree that the empirical web deviates from the null hypothesis. We used two randomizations: “standard,” in which all links are shuffled, with the restriction that single-directional and bidirectional links are only shuffled with each other [11], and “compartmented,” which proceeds in the same fashion but with the additional restriction that links are shuffled only with those of the same type (links between free-living taxa, between parasites and free-living hosts, etc.). For a given web, we quantified the motif structure with a vector of *z*-scores *Z* = {*z_i_*}, which has one component for each of the 13 three-species motifs. To compare webs, we plotted the normalized profile, the vector of *z*-scores normalized to length 1. This aids in graphical comparison because larger and more densely connected webs tend to exhibit more pronounced patterns of motif representation. The occurrence of motifs in empirical webs was compared to niche model expectations.

We used a probabilistic niche model (Box 1) [63],[64] based on maximum likelihood methods [16] to parameterize the niche model directly against each empirical food web. The probabilistic niche model tests the overall model fit to the data rather than to partial aspects of structure. It produces a MLE of the niche model parameters for each species *i* in a given web: its niche position *n_i_*, position of feeding range *c_i_*, and feeding range (or “trophic niche”) *r_i_*. This allows computation of the probability of each link in a web according to the model, and the overall expected fraction of links (*f*_L_) in a web predicted correctly by the model (Table 1, Metric 22). The one-dimensional probabilistic niche model outperforms [64] other recently proposed structural models [15],[16]. We calculated *f*_L_ for one- and two-dimensional versions of the model and compared their performance for each web using the Akaike Information Criterion [93]. The MLE parameter sets were used to explore the trophic niche structure of parasite and free-living species.

## Supporting Information

Figure S1 **Cumulative resource distributions.** The cumulative degree distributions for links to resources are presented in log-linear format. The link data are normalized (divided) by the mean number of links per species (*L/S*) in each web. The seven food webs are Bahia Falsa (Fals), Carpinteria Salt Marsh (Carp), Estero de Punta Banda (Punt), Flensburg Fjord (Flens), Otago Harbor (Otag), Sylt Tidal Basin (Sylt), and Ythan Estuary (Ythan).
(TIF)

Figure S2 **Cumulative consumer distributions.** The cumulative degree distributions for links to consumers are presented in log-linear format. The link data are normalized (divided) by the mean number of links per species (*L/S*) in each web. See Figure S1 legend for food web names.
(TIF)

Figure S3 **Motif analysis using compartmented randomization.** The representation of three-node motifs in three versions each of seven food webs. (A) Results for webs with free-living taxa only. (B) Results for webs with parasites but not concomitant predation links. (C) Results for webs with parasites and concomitant predation links. Motif labels and graphics are shown at the top of the figure, with arrowheads pointing from resources to consumers. The data points show the normalized profile overrepresentation (>0) or underrepresentation (<0) of each motif in the seven food webs. The grey bars represent predictions of the niche model for overrepresentation (>0) or underrepresentation (<0) of the individual motifs.
(TIF)

Figure S4 **Visualization of trophic niches of species in the Bahia Falsa and Carpinteria Salt Marsh webs.** Empirically observed links, organized by the probabilistic niche model MLE values for consumer niche position (*c*) and resource niche value (*n*), for Bahia Falsa (Fals) and Carpinteria Salt Marsh (Carp). “Free” refers to webs with free-living species only; “Par” refers to webs with parasites but not concomitant links; “ParCon” refers to webs with parasites and concomitant links. The links to resources of free-living taxa are red, and those of parasite taxa are blue.
(TIF)

Figure S5 **Visualization of trophic niches of species in the Otago Harbor and Sylt Tidal Basin webs.** Empirically observed links, organized by the probabilistic niche model MLE values for consumer niche position (*c*) and resource niche value (*n*), for Otago Harbor (Otag) and Sylt Tidal Basin (Sylt). “Free” refers to webs with free-living species only; “Par” refers to webs with parasites but not concomitant links; “ParCon” refers to webs with parasites and concomitant links. The links to resources of free-living taxa are red, and those of parasite taxa are blue.
(TIF)

Figure S6 **Visualization of trophic niches of species in the Flensburg Fjord and Ythan Estuary webs.** Empirically observed links, organized by the probabilistic niche model MLE values for consumer niche position (*c*) and resource niche value (*n*), for Flensburg Fjord (Flens) and Ythan Estuary (Ythan). “Free” refers to webs with free-living species only; “Par” refers to webs with parasites but not concomitant links; “ParCon” refers to webs with parasites and concomitant links. The links to resources of free-living taxa are red, and those of parasite taxa are blue.
(TIF)

Methods S1 **Additional references associated with the 28 previously studied food webs in Table S5.**
(DOCX)

Table S1 **Basic properties of the original species food webs.** Fals, Carp, Punt, Flens, Otag, Sylt, and Ythan refer to the food webs for Bahia Falsa, Carpinteria Salt Marsh, Estero de Punta Banda, Flensburg Fjord, Otago Harbor, Sylt Tidal Basin, and Ythan Estuary, respectively. “Free” refers to webs with free-living species only; “Par” refers to webs with parasites but not concomitant links, and “ParCon” refers to webs with parasites and concomitant links. *S*, *L*, *L/*S, *C*, and *C*_adj_ are defined in Table 1 (Metrics 1–5). *S*_Free_, *S*_Par_, and *S*_Bas_ refer to the fraction of taxa that are free-living, parasite, and basal, respectively.
(DOCX)

Table S2 **Number of links by type for trophic species webs.** Refer to Table S1 for food web naming conventions. *L* refers to number of trophic links, *L*_FL_ refers to number of links involving a free-living species, *L*_Par_ refers to number of links involving a parasite, FL-FL refers to links between free-living species, Par-FL refers to parasite–host links, Par-Par refers to links between parasites, and FL-Par refers to links where parasites are consumed by free-living species.
(DOCX)

Table S3 **Number of links by type for original species webs.** Refer to Table S1 for food web naming conventions. *L* refers to number of trophic links, *L*_FL_ refers to number of links involving a free-living species, *L*_Par_ refers to number of links involving a parasite, FL-FL refers to links between free-living species, Par-FL refers to parasite–host links, Par-Par refers to links between parasites, and FL-Par refers to links where parasites are consumed by free-living species.
(DOCX)

Table S4 **Degree distribution results for the MaxEnt model.** Refer to Table S1 for food web naming conventions. “Cons” refers to consumer distribution. “Res” refers to resource distribution. ƒ_G_ is goodness of fit, where ƒ_G_≤0.95 indicates that the empirical web's degree distribution is not significantly different from the model distribution at the 95% confidence interval. A significant difference in ƒ_G_ indicates an offset of the empirical distributions compared to the MaxEnt distribution. *W*_95_ is relative width of the degree distribution, where −1≤*W*_95_≤1 indicates that the empirical distribution is neither significantly narrower (*W*_95_<−1) nor significantly broader (*W*_95_>1) than the distribution predicted by the model at the 95% confidence interval. Bold indicates ƒ_G_ or *W*_95_ values that differ significantly from model expectations.
(DOCX)

Table S5 **Basic properties of 28 previously studied food webs used for scale dependence analyses.** *S*, *L*, *L/S*, and *C* are defined in Table 1 (Metrics 1–4). An “x” indicates the subset of ten webs utilized in analyses of scale dependence of absolute niche ME (|ME|) [12]. All 28 webs were used in assessments of relative width of the consumer distribution (*W*_95 Cons_) and fraction of links correctly predicted by the probabilistic niche model (*f*_L_). The 28 webs represent a subset of overlapping webs from [62],[64], with the following webs eliminated: webs with *S*<25, source webs, replicate webs from a particular habitat, and earlier versions of current webs. Additional references given in Methods S1. Where “E” followed by a number appears in parentheses following a web name, it refers to the ECOWeB number for that web [97].
(DOCX)

Table S6 **Niche model errors for types of taxa.** See Table S1 for food web naming conventions. The values show the niche MEs for properties related to types of species in the web. Network structure properties are described in Table 1 (Metrics 6–12). Values of ME>|1| are shown in bold and indicate a poor fit of the niche model prediction to the empirical value. Negative MEs indicate niche model underestimation of the empirical value; positive MEs indicate niche model overestimation of the empirical value.
(DOCX)

Table S7 **Niche model errors for web structure properties.** See Table S1 for food web naming conventions. The values show the niche MEs for properties related to types of species in the web. The properties are defined in Table 1 (Metrics 13–19). Values of ME>|1| are shown in bold and indicate a poor fit of the niche model prediction to the empirical value. Negative MEs indicate niche model underestimation of the empirical value; positive MEs indicate niche model overestimation of the empirical value.
(DOCX)

Table S8 **Probabilistic niche model results.** See Table S1 for food web naming conventions. *f*_L-1D_ and *f*_L-2D_ indicate the fraction of links in an empirical web predicted correctly by the one-dimensional and two-dimensional versions of the probabilistic niche model (Box 1), respectively. AIC-1D and AIC-2D give the Akaike Information Criterion values [93] for the performance of the one-dimensional and two-dimensional versions of the probabilistic niche model.
(DOCX)

## Abbreviations

FL: free-living species
ME: model error
MLE: maximum likelihood estimate
Par: parasite species
