# Supplementary figures and images for: Parasites Affect Food Web Structure Primarily through Increased Diversity and Complexity
Source: PLoS Biol. 2013 Jun 11;11(6):e1001579. doi: 10.1371/journal.pbio.1001579 (PMC3679000; doi:10.1371/journal.pbio.1001579)

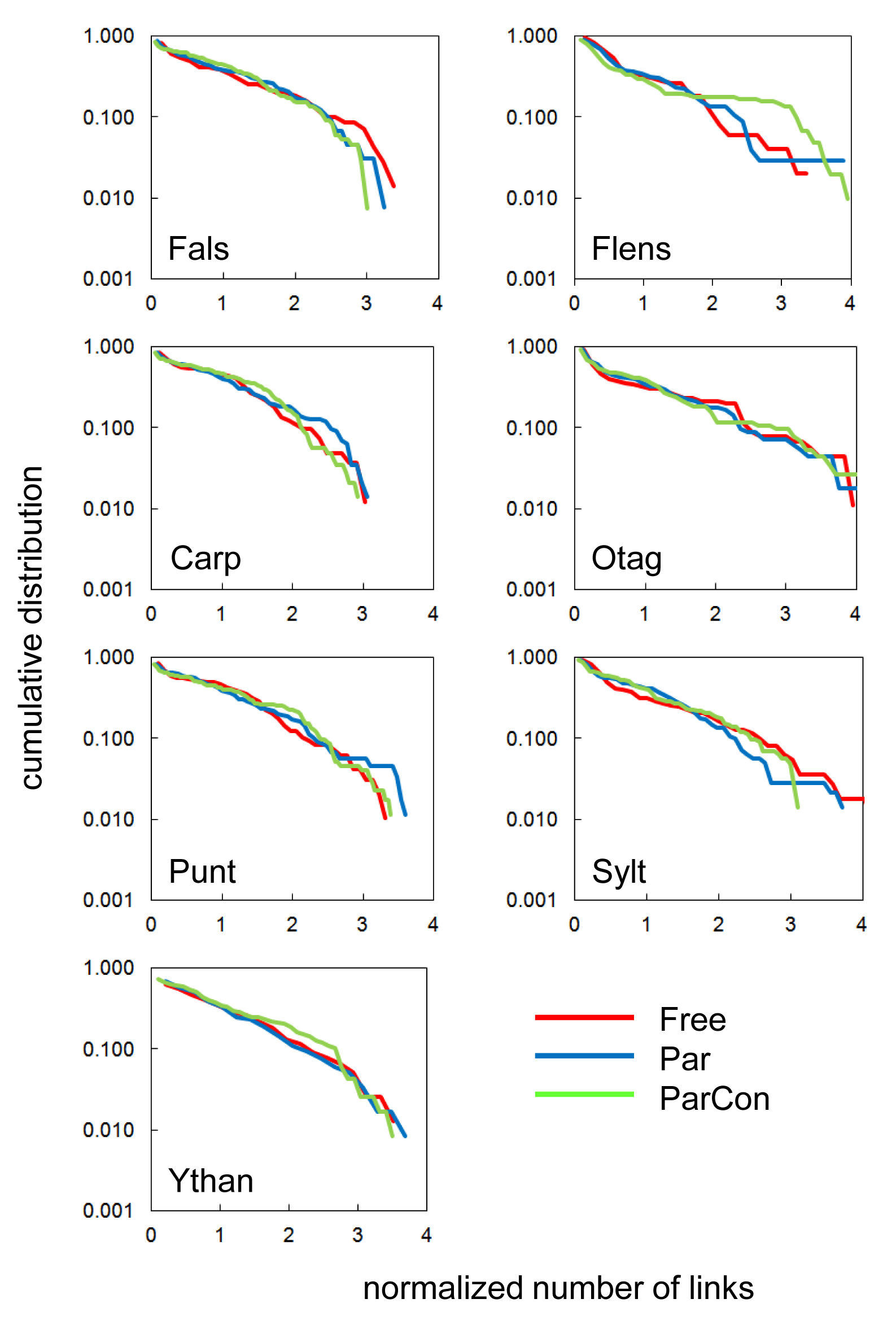

Supplement: Figure S1 — Cumulative resource distributions. The cumulative degree distributions for links to resources are presented in log-linear format. The link data are normalized (divided) by the mean number of links per species (L/S) in each web. The seven food webs are Bahia Falsa (Fals), Carpinteria Salt Marsh (Carp), Estero de Punta Banda (Punt), Flensburg Fjord (Flens), Otago Harbor (Otag), Sylt Tidal Basin (Sylt), and Ythan Estuary (Ythan). (TIF) [file pbio.1001579.s001.tif]

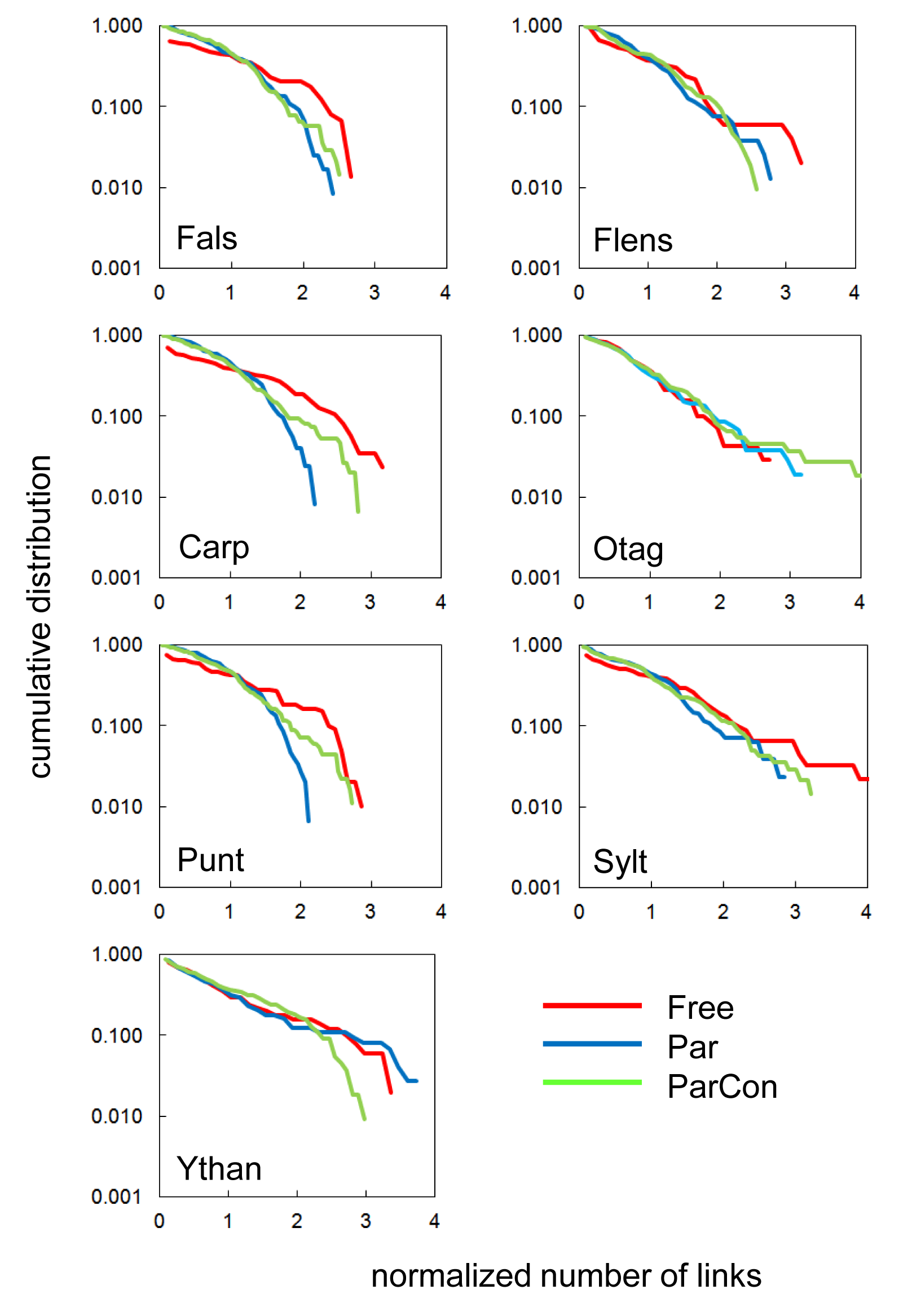

Supplement: Figure S2 — Cumulative consumer distributions. The cumulative degree distributions for links to consumers are presented in log-linear format. The link data are normalized (divided) by the mean number of links per species (L/S) in each web. See Figure S1 legend for food web names. (TIF) [file pbio.1001579.s002.tif]

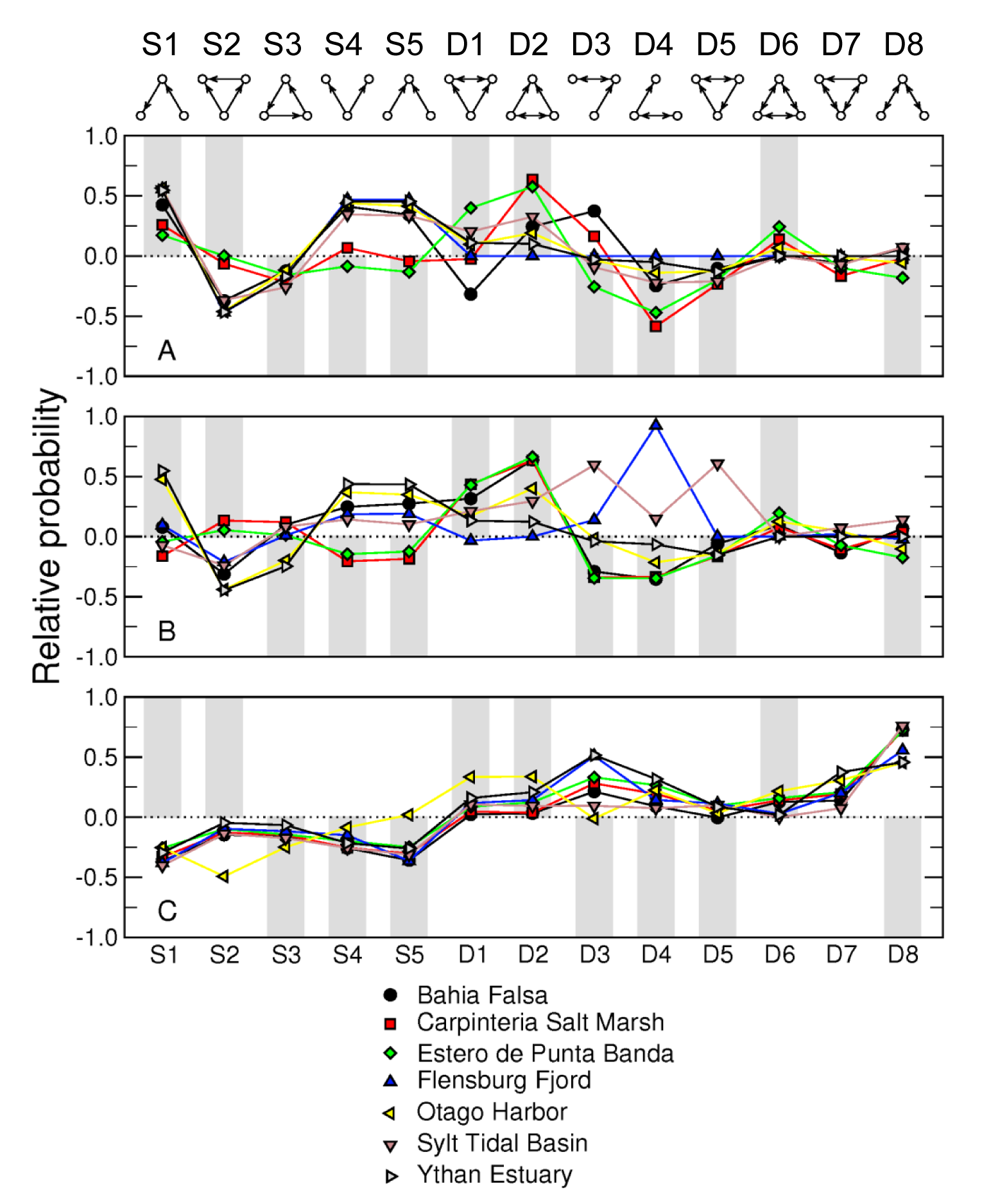

Supplement: Figure S3 — Motif analysis using compartmented randomization. The representation of three-node motifs in three versions each of seven food webs. (A) Results for webs with free-living taxa only. (B) Results for webs with parasites but not concomitant predation links. (C) Results for webs with parasites and concomitant predation links. Motif labels and graphics are shown at the top of the figure, with arrowheads pointing from resources to consumers. The data points show the normalized profile overrepresentation (>0) or underrepresentation (<0) of each motif in the seven food webs. The grey bars represent predictions of the niche model for overrepresentation (>0) or underrepresentation (<0) of the individual motifs. (TIF) [file pbio.1001579.s003.tif]

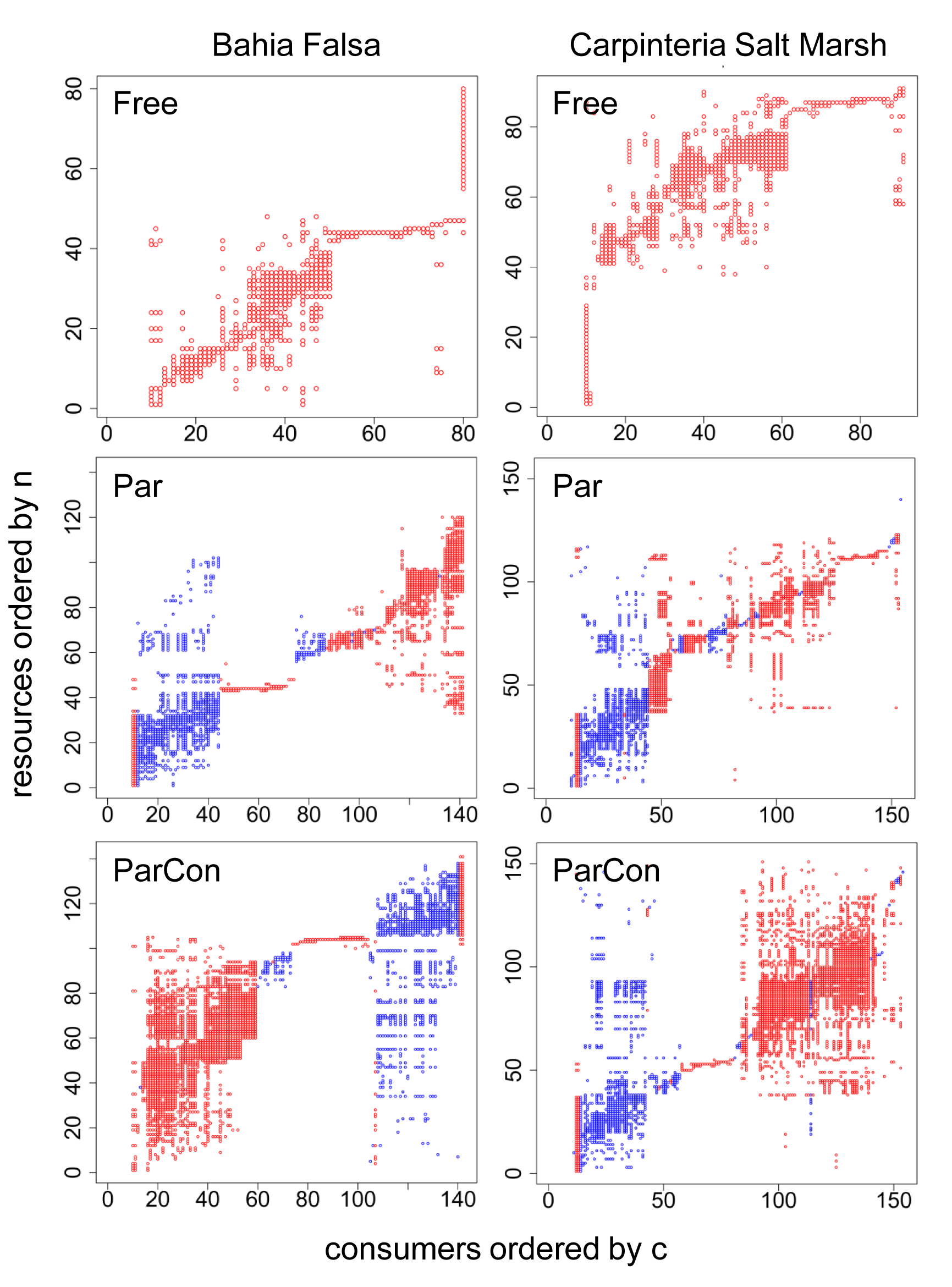

Supplement: Figure S4 — Visualization of trophic niches of species in the Bahia Falsa and Carpinteria Salt Marsh webs. Empirically observed links, organized by the probabilistic niche model MLE values for consumer niche position (c) and resource niche value (n), for Bahia Falsa (Fals) and Carpinteria Salt Marsh (Carp). “Free” refers to webs with free-living species only; “Par” refers to webs with parasites but not concomitant links; “ParCon” refers to webs with parasites and concomitant links. The links to resources of free-living taxa are red, and those of parasite taxa are blue. (TIF) [file pbio.1001579.s004.tif]

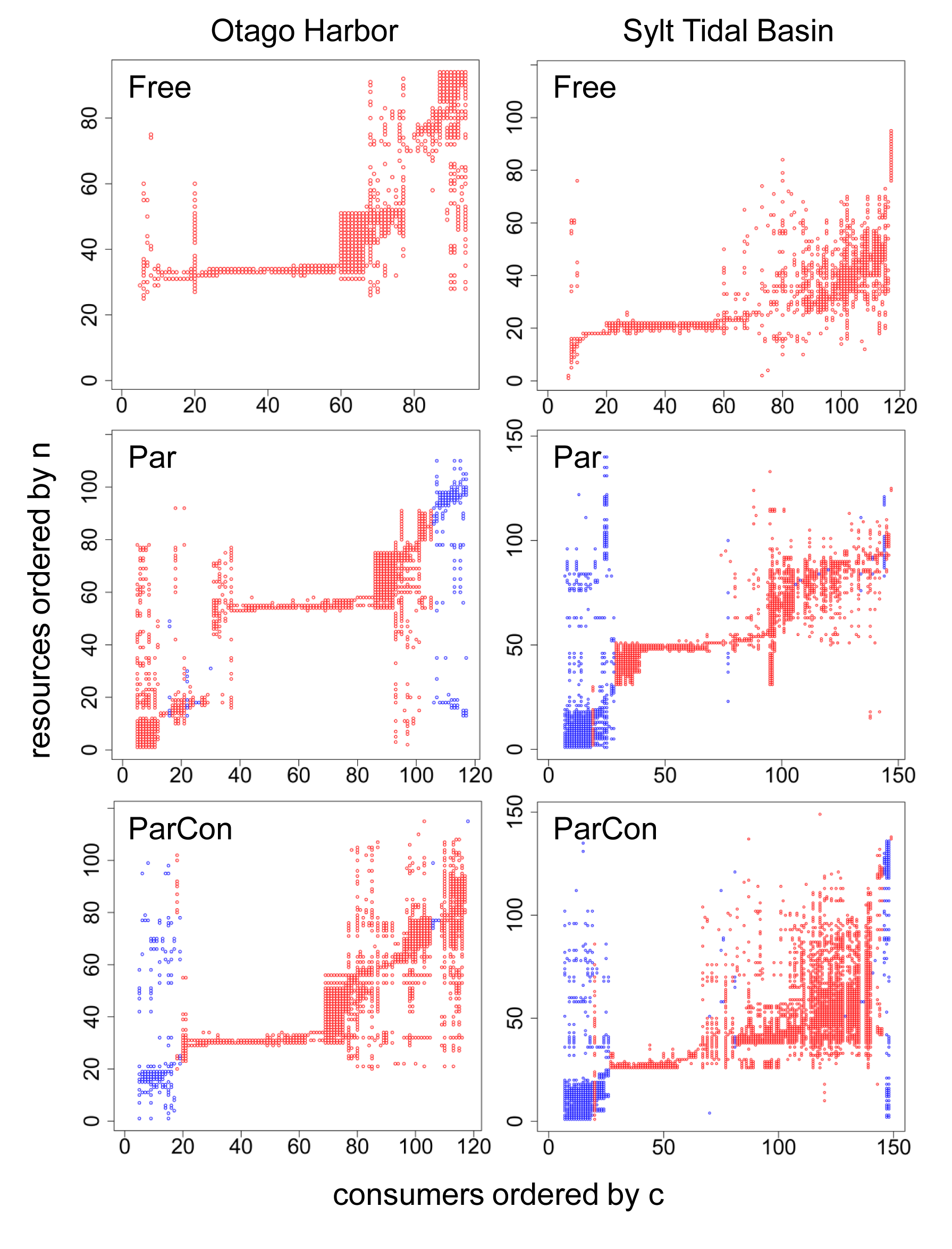

Supplement: Figure S5 — Visualization of trophic niches of species in the Otago Harbor and Sylt Tidal Basin webs. Empirically observed links, organized by the probabilistic niche model MLE values for consumer niche position (c) and resource niche value (n), for Otago Harbor (Otag) and Sylt Tidal Basin (Sylt). “Free” refers to webs with free-living species only; “Par” refers to webs with parasites but not concomitant links; “ParCon” refers to webs with parasites and concomitant links. The links to resources of free-living taxa are red, and those of parasite taxa are blue. (TIF) [file pbio.1001579.s005.tif]

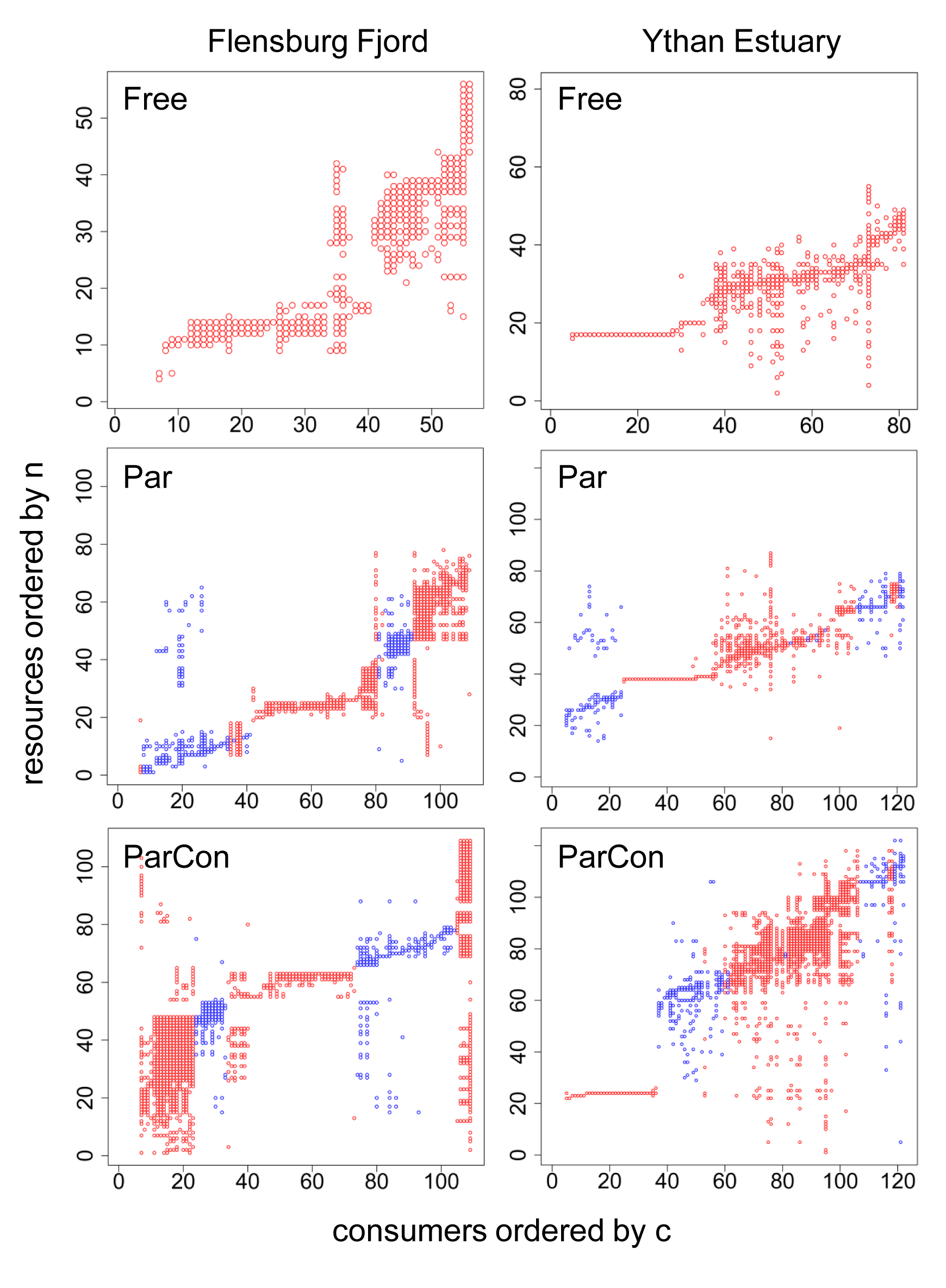

Supplement: Figure S6 — Visualization of trophic niches of species in the Flensburg Fjord and Ythan Estuary webs. Empirically observed links, organized by the probabilistic niche model MLE values for consumer niche position (c) and resource niche value (n), for Flensburg Fjord (Flens) and Ythan Estuary (Ythan). “Free” refers to webs with free-living species only; “Par” refers to webs with parasites but not concomitant links; “ParCon” refers to webs with parasites and concomitant links. The links to resources of free-living taxa are red, and those of parasite taxa are blue. (TIF) [file pbio.1001579.s006.tif]
