# Supplementary material for: Parasites Affect Food Web Structure Primarily through Increased Diversity and Complexity
Source: PLoS Biol. 2013 Jun 11;11(6):e1001579. doi: 10.1371/journal.pbio.1001579 (PMC3679000; doi:10.1371/journal.pbio.1001579)
Supplement: Methods S1 — Additional references associated with the 28 previously studied food webs in Table S5. (DOCX) [file pbio.1001579.s007.docx]

**Methods S1. Additional References Associated with 28 Food Webs in Table S5**

1. Havens K. (1992) Scale and structure in natural food webs. Science 257: 1107–1109.
2. Warren PH. (1989) Spatial and temporal variation in the structure of a freshwater food web. Oikos 55: 299–311.
3. Yodzis P. (1998) Local trophodynamics and the interaction of marine mammals and fisheries in the Benguela ecosystem. J Anim Ecolo 67: 635-658.
4. Polis GA. (1991) Complex trophic interactions in deserts: an empirical critique of food-web theory. Amer Nat 138: 123-155.
5. Tavares-Cromar AF, Williams DD. (1996) The importance of temporal resolution in food web analysis: Evidence from a detritus-based stream. Ecol Monog 66: 91–113.
6. Closs GP, Lake PS. (1994) Spatial and temporal variation in the structure of an intermittent stream food web. Ecol Monog 64: 1–21.
7. Baird D, Ulanowicz RE. (1989) The seasonal dynamics of the Chesapeake Bay ecosystem. Ecol Monog 59: 329–364.
8. Goldwasser L, Roughgarden JA. (1993) Construction of a large Caribbean food web. Ecology 74: 1216–1233.
9. Thompson RM, Townsend CR. (2003) Impacts on stream food webs of native and exotic forest: an intercontinental comparison. Ecology 84: 145–161
10. Christian RR, Luczkovich JJ. (1999) Organizing and understanding a winter's seagrass foodweb network through effective trophic levels. Ecol Model 117: 99–124.
11. Opitz S. (1996) Trophic Interactions in Caribbean Coral Reefs. ICLARM Technical Report 43, Manila.
12. Thompson RM, Townsend CR. (2004) Landuse influences on New Zealand stream communities – effects on species composition, functional organization and food-web structure. New Zeal J Mar Freshwater Res 38: 595-608.
13. Link J. (2002) Does food web theory work for marine ecosystems? Mar Ecol Prog Ser 230: 1-9.
14. Waide RB, Reagan WB. (1996) The Food Web of a Tropical Rainforest. Chicago: University of Chicago Press.
