# Supplementary material for: Parasites Affect Food Web Structure Primarily through Increased Diversity and Complexity
Source: PLoS Biol. 2013 Jun 11;11(6):e1001579. doi: 10.1371/journal.pbio.1001579 (PMC3679000; doi:10.1371/journal.pbio.1001579)
Supplement: Table S1 — Basic properties of the original species food webs. Fals, Carp, Punt, Flens, Otag, Sylt, and Ythan refer to the food webs for Bahia Falsa, Carpinteria Salt Marsh, Estero de Punta Banda, Flensburg Fjord, Otago Harbor, Sylt Tidal Basin, and Ythan Estuary, respectively. “Free” refers to webs with free-living species only; “Par” refers to webs with parasites but not concomitant links, and “ParCon” refers to webs with parasites and concomitant links. S, L, L/S, C, and C adj are defined in Table 1 (Metrics 1–5). S Free, S Par, and S Bas refer to the fraction of taxa that are free-living, parasite, and basal, respectively. (DOCX) [file pbio.1001579.s008.docx]

**Table S1. Basic Properties of Original Species Food Webs**

|  | *S* | *L* | *L/S* | *C* | *C_adj_* | *S*_Free_ | *S*_Par_ | *S*_Bas_ |
| --- | --- | --- | --- | --- | --- | --- | --- | --- |
| FalsFree | 119 | 1077 | 9.05 | 0.076 |  | 1.00 | 0.00 | 0.14 |
| FalsPar | 171 | 2234 | 13.06 | 0.076 | 0.110 | 0.70 | 0.30 | 0.10 |
| FalsParCon | 171 | 3720 | 21.75 | 0.127 |  | 0.70 | 0.30 | 0.10 |
| CarpFree | 107 | 970 | 9.07 | 0.085 |  | 1.00 | 0.00 | 0.11 |
| CarpPar | 165 | 2187 | 13.25 | 0.080 | 0.124 | 0.65 | 0.35 | 0.07 |
| CarpParCon | 165 | 3708 | 22.47 | 0.136 |  | 0.65 | 0.35 | 0.07 |
| PuntFree | 138 | 1657 | 12.01 | 0.087 |  | 1.00 | 0.00 | 0.10 |
| PuntPar | 214 | 3334 | 15.58 | 0.073 | 0.113 | 0.64 | 0.36 | 0.07 |
| PuntParCon | 214 | 5653 | 26.42 | 0.123 |  | 0.64 | 0.36 | 0.07 |
| FlensFree | 77 | 579 | 7.52 | 0.098 |  | 1.00 | 0.00 | 0.08 |
| FlensPar | 123 | 968 | 7.87 | 0.064 | 0.102 | 0.63 | 0.37 | 0.05 |
| FlensParCon | 123 | 1406 | 11.43 | 0.093 |  | 0.63 | 0.37 | 0.05 |
| OtagFree | 123 | 1206 | 9.80 | 0.080 |  | 1.00 | 0.00 | 0.02 |
| OtagPar | 142 | 1487 | 10.47 | 0.074 | 0.085 | 0.87 | 0.13 | 0.02 |
| OtagParCon | 142 | 1844 | 12.99 | 0.091 |  | 0.87 | 0.13 | 0.02 |
| SyltFree | 126 | 1052 | 8.35 | 0.066 |  | 1.00 | 0.00 | 0.05 |
| SyltPar | 161 | 1950 | 12.11 | 0.075 | 0.096 | 0.78 | 0.22 | 0.04 |
| SyltParCon | 161 | 3005 | 18.66 | 0.116 |  | 0.78 | 0.22 | 0.04 |
| YthanFree | 91 | 420 | 4.62 | 0.051 |  | 1.00 | 0.00 | 0.04 |
| YthanPar | 133 | 597 | 4.49 | 0.034 | 0.049 | 0.68 | 0.32 | 0.03 |
| YthanParCon | 133 | 1391 | 10.46 | 0.079 |  | 0.68 | 0.32 | 0.03 |
