# Supplementary material for: Parasites Affect Food Web Structure Primarily through Increased Diversity and Complexity
Source: PLoS Biol. 2013 Jun 11;11(6):e1001579. doi: 10.1371/journal.pbio.1001579 (PMC3679000; doi:10.1371/journal.pbio.1001579)
Supplement: Table S2 — Number of links by type for trophic species webs. Refer to Table S1 for food web naming conventions. L refers to number of trophic links, L FL refers to number of links involving a free-living species, L Par refers to number of links involving a parasite, FL-FL refers to links between free-living species, Par-FL refers to parasite–host links, Par-Par refers to links between parasites, and FL-Par refers to links where parasites are consumed by free-living species. (DOCX) [file pbio.1001579.s009.docx]

**Table S2. Number of Links by Type, Trophic Species Webs**

| Food Web-Type | *L* | *L_FL_* | *L_Par_* | FL-FL | Par-FL | Par-Par | FL-Par |
| --- | --- | --- | --- | --- | --- | --- | --- |
| Fals-Free | 527 | 527 | 0 | 527 | 0 | 0 | 0 |
| Fals-Par | 1792 | 1627 | 1034 | 758 | 687 | 165 | 182 |
| Fals-ParCon | 3006 | 2841 | 2239 | 767 | 687 | 165 | 1387 |
| Carp-Free | 761 | 761 | 0 | 761 | 0 | 0 | 0 |
| Carp-Par | 1982 | 1816 | 1120 | 862 | 681 | 166 | 273 |
| Carp-ParCon | 3350 | 3184 | 2480 | 870 | 681 | 166 | 1633 |
| Punt-Free | 1085 | 1085 | 0 | 1085 | 0 | 0 | 0 |
| Punt-Par | 2838 | 2669 | 1499 | 1339 | 732 | 169 | 598 |
| Punt-ParCon | 4671 | 4502 | 3323 | 1348 | 732 | 169 | 2422 |
| Flens-Free | 358 | 358 | 0 | 358 | 0 | 0 | 0 |
| Flens-Par | 846 | 811 | 326 | 520 | 227 | 35 | 64 |
| Flens-ParCon | 1252 | 1217 | 732 | 520 | 227 | 35 | 470 |
| Otag-Free | 751 | 751 | 0 | 751 | 0 | 0 | 0 |
| Otag-Par | 1054 | 1035 | 236 | 818 | 136 | 19 | 81 |
| Otag-ParCon | 1354 | 1335 | 536 | 818 | 145 | 19 | 372 |
| Sylt-Free | 993 | 993 | 0 | 993 | 0 | 0 | 0 |
| Sylt-Par | 1708 | 1654 | 711 | 997 | 479 | 54 | 178 |
| Sylt-ParCon | 2680 | 2619 | 1683 | 997 | 495 | 61 | 1127 |
| Ythan-Free | 394 | 394 | 0 | 394 | 0 | 0 | 0 |
| Ythan-Par | 576 | 576 | 170 | 406 | 170 | 0 | 0 |
| Ythan-ParCon | 1284 | 1200 | 878 | 406 | 170 | 84 | 624 |
