# Supplementary material for: Parasites Affect Food Web Structure Primarily through Increased Diversity and Complexity
Source: PLoS Biol. 2013 Jun 11;11(6):e1001579. doi: 10.1371/journal.pbio.1001579 (PMC3679000; doi:10.1371/journal.pbio.1001579)
Supplement: Table S3 — Number of links by type for original species webs. Refer to Table S1 for food web naming conventions. L refers to number of trophic links, L FL refers to number of links involving a free-living species, L Par refers to number of links involving a parasite, FL-FL refers to links between free-living species, Par-FL refers to parasite–host links, Par-Par refers to links between parasites, and FL-Par refers to links where parasites are consumed by free-living species. (DOCX) [file pbio.1001579.s010.docx]

**Table S3. Number of Links by Type, Original Species Webs**

| Food Web-Type | *L* | *L_FL_* | *L_Par_* | FL-FL | Par-FL | Par-Par | FL-Par |
| --- | --- | --- | --- | --- | --- | --- | --- |
| Fals-Free | 1077 | 1077 | 0 | 1077 | 0 | 0 | 0 |
| Fals-Par | 2234 | 2069 | 1157 | 1077 | 807 | 165 | 185 |
| Fals-ParCon | 3720 | 3555 | 2643 | 1086 | 807 | 165 | 1662 |
| Carp-Free | 970 | 970 | 0 | 970 | 0 | 0 | 0 |
| Carp-Par | 2187 | 2021 | 1217 | 970 | 755 | 166 | 296 |
| Carp-ParCon | 3708 | 3542 | 2738 | 978 | 755 | 166 | 1809 |
| Punt-Free | 1657 | 1657 | 0 | 1657 | 0 | 0 | 0 |
| Punt-Par | 3334 | 3165 | 1677 | 1657 | 835 | 169 | 673 |
| Punt-ParCon | 5653 | 5484 | 3996 | 1668 | 835 | 169 | 2981 |
| Flens-Free | 579 | 579 | 0 | 579 | 0 | 0 | 0 |
| Flens-Par | 968 | 928 | 389 | 579 | 271 | 40 | 78 |
| Flens-ParCon | 1406 | 1366 | 827 | 579 | 271 | 40 | 516 |
| Otag-Free | 1206 | 1206 | 0 | 1206 | 0 | 0 | 0 |
| Otag-Par | 1487 | 1468 | 281 | 1206 | 173 | 19 | 89 |
| Otag-ParCon | 1844 | 1825 | 638 | 1206 | 173 | 19 | 446 |
| Sylt-Free | 1052 | 1052 | 0 | 1052 | 0 | 0 | 0 |
| Sylt-Par | 1950 | 1880 | 898 | 1052 | 552 | 70 | 276 |
| Sylt-ParCon | 3005 | 2935 | 1953 | 1052 | 552 | 70 | 1331 |
| Ythan-Free | 420 | 420 | 0 | 420 | 0 | 0 | 0 |
| Ythan-Par | 597 | 597 | 177 | 420 | 177 | 0 | 0 |
| Ythan-ParCon | 1391 | 1272 | 971 | 420 | 177 | 119 | 675 |
