# Supplementary material for: Parasites Affect Food Web Structure Primarily through Increased Diversity and Complexity
Source: PLoS Biol. 2013 Jun 11;11(6):e1001579. doi: 10.1371/journal.pbio.1001579 (PMC3679000; doi:10.1371/journal.pbio.1001579)
Supplement: Table S4 — Degree distribution results for the MaxEnt model. Refer to Table S1 for food web naming conventions. “Cons” refers to consumer distribution. “Res” refers to resource distribution. ƒG is goodness of fit, where ƒG≤0.95 indicates that the empirical web's degree distribution is not significantly different from the model distribution at the 95% confidence interval. A significant difference in ƒG indicates an offset of the empirical distributions compared to the MaxEnt distribution. W 95 is relative width of the degree distribution, where −1≤W 95≤1 indicates that the empirical distribution is neither significantly narrower (W 95<−1) nor significantly broader (W 95>1) than the distribution predicted by the model at the 95% confidence interval. Bold indicates ƒG or W 95 values that differ significantly from model expectations. (DOCX) [file pbio.1001579.s011.docx]

**Table S4. Degree Distribution Results for the MaxEnt Model**

| Food Web-Type | ƒ_G Cons_ | W_95 Cons_ | ƒ_G Res_ | W_95 Res_ |
| --- | --- | --- | --- | --- |
| Fals-Free | **1.000** | 0.08 | 0.846 | 0.34 |
| Fals-Par | 0.879 | **-1.83** | 0.881 | -0.02 |
| Fals-ParCon | 0.894 | **-2.18** | **0.999** | -0.22 |
| Carp-Free | **0.983** | 0.23 | 0.910 | -0.23 |
| Carp-Par | 0.933 | **-2.29** | **0.978** | -0.24 |
| Carp-ParCon | 0.688 | **-1.89** | **1.000** | -0.45 |
| Punt-Free | **1.000** | -0.15 | 0.946 | -0.07 |
| Punt-Par | **0.953** | **-2.85** | **1.000** | 0.06 |
| Punt-ParCon | 0.737 | **-2.12** | **1.000** | 0.15 |
| Flens-Free | 0.794 | -0.16 | **0.964** | -0.10 |
| Flens-Par | 0.324 | **-1.35** | 0.932 | 0.19 |
| Flens-ParCon | **0.966** | **-1.34** | **1.000** | **1.28** |
| Otag-Free | 0.816 | -0.26 | **1.000** | 0.90 |
| Otag-Par | 0.826 | -0.24 | **1.000** | 0.52 |
| Otag-ParCon | 0.409 | -0.44 | **1.000** | 0.60 |
| Sylt-Free | **0.998** | 0.32 | **0.988** | 0.51 |
| Sylt-Par | 0.762 | **-1.02** | **0.988** | 0.05 |
| Sylt-ParCon | 0.426 | -0.88 | **0.996** | -0.08 |
| Ythan-Free | 0.351 | 0.28 | 0.899 | 0.74 |
| Ythan-Par | 0.749 | 0.38 | 0.904 | 0.73 |
| Ythan-ParCon | 0.366 | -0.31 | **0.999** | 0.43 |
