# Supplementary material for: Parasites Affect Food Web Structure Primarily through Increased Diversity and Complexity
Source: PLoS Biol. 2013 Jun 11;11(6):e1001579. doi: 10.1371/journal.pbio.1001579 (PMC3679000; doi:10.1371/journal.pbio.1001579)
Supplement: Table S5 — Basic properties of 28 previously studied food webs used for scale dependence analyses. S, L, L/S, and C are defined in Table 1 (Metrics 1–4). An “x” indicates the subset of ten webs utilized in analyses of scale dependence of absolute niche ME (|ME|) [12]. All 28 webs were used in assessments of relative width of the consumer distribution (W 95 Cons) and fraction of links correctly predicted by the probabilistic niche model (f L). The 28 webs represent a subset of overlapping webs from [62],[64], with the following webs eliminated: webs with S<25, source webs, replicate webs from a particular habitat, and earlier versions of current webs. Additional references given in Methods S1. Where “E” followed by a number appears in parentheses following a web name, it refers to the ECOWeB number for that web [97]. (DOCX) [file pbio.1001579.s012.docx]

**Table S5. Basic Properties of 28 Food Webs Used for Scale Dependence Analyses**

| Web Name | *S* | *L* | *L/S* | *C* | \|ME\| | Citation |
| --- | --- | --- | --- | --- | --- | --- |
| Bridge Brook Lake | 25 | 107 | 4.28 | 0.171 | x | 98 |
| Skipwith Pond | 25 | 197 | 7.88 | 0.315 | x | 99 |
| Deep Creek (E 207) | 26 | 97 | 3.73 | 0.143 |  | 97 |
| Benguela Marine | 29 | 203 | 7.00 | 0.241 | x | 100 |
| Crocodile Creek (E 33) | 29 | 48 | 1.66 | 0.057 |  | 97 |
| Trelase Woods (E 59) | 29 | 61 | 2.10 | 0.073 |  | 97 |
| Coachella Valley | 29 | 262 | 9.03 | 0.312 | x | 101 |
| Duffin Creek | 30 | 118 | 3.93 | 0.131 |  | 102 |
| Lerderderg Stream | 31 | 61 | 1.97 | 0.063 |  | 103 |
| Chesapeake Bay | 31 | 69 | 2.23 | 0.072 | x | 104 |
| Lake Nyasa Rocky Shore (E 38) | 31 | 95 | 3.07 | 0.099 |  | 97 |
| Lake Nyasa Sandy Shore (E 39) | 33 | 70 | 2.12 | 0.064 |  | 97 |
| Monterey Bay Rocky Shore (E 106) | 35 | 73 | 2.09 | 0.060 |  | 97 |
| St. Martin Island | 42 | 205 | 4.88 | 0.116 | x | 105 |
| Coweeta Stream | 47 | 109 | 2.32 | 0.049 |  | 106 |
| St. Marks Estuary | 48 | 221 | 4.60 | 0.096 | x | 107 |
| Sonora Desert (E 99) | 48 | 138 | 2.88 | 0.060 |  | 97 |
| Air, Nidd, Wharf Stream Bed (E 210) | 49 | 142 | 2.90 | 0.059 |  | 97 |
| Caribbean Reef | 50 | 556 | 11.12 | 0.222 | x | 108 |
| Oak Gall (E 167) | 54 | 174 | 3.22 | 0.060 |  | 97 |
| Troy Stream | 63 | 160 | 2.54 | 0.040 |  | 106 |
| Sutton Stream | 67 | 312 | 4.66 | 0.070 |  | 109 |
| N.E. U.S. Shelf Marine | 79 | 1400 | 18.08 | 0.229 | x | 110 |
| Little Rock Lake | 92 | 997 | 10.84 | 0.118 | x | 70 |
| Canton Creek | 102 | 697 | 6.83 | 0.067 |  | 109 |
| Shortgrass Prairie (E 123) | 106 | 379 | 3.58 | 0.034 |  | 97 |
| Stony Stream | 109 | 829 | 7.61 | 0.070 |  | 109 |
| El Verde Rainforest | 155 | 1509 | 9.74 | 0.063 |  | 111 |
