# Supplementary material for: Parasites Affect Food Web Structure Primarily through Increased Diversity and Complexity
Source: PLoS Biol. 2013 Jun 11;11(6):e1001579. doi: 10.1371/journal.pbio.1001579 (PMC3679000; doi:10.1371/journal.pbio.1001579)
Supplement: Table S6 — Niche model errors for types of taxa. See Table S1 for food web naming conventions. The values show the niche MEs for properties related to types of species in the web. Network structure properties are described in Table 1 (Metrics 6–12). Values of ME>|1| are shown in bold and indicate a poor fit of the niche model prediction to the empirical value. Negative MEs indicate niche model underestimation of the empirical value; positive MEs indicate niche model overestimation of the empirical value. (DOCX) [file pbio.1001579.s013.docx]

**Table S6. Niche Model Errors for Types of Taxa**

| Food Web-Type | Top | Int | Bas | Herb | Omn | Can | Loop |
| --- | --- | --- | --- | --- | --- | --- | --- |
| Fals-Free | -0.25 | -0.33 | 0.80 | **-4.20** | **1.63** | **1.50** | **-3.17** |
| Fals-Par | **-1.42** | 0.73 | 1.00 | **-4.80** | **1.70** | **2.00** | **-5.50** |
| Fals-ParCon | -0.11 | 0.00 | 0.00 | **-7.00** | **3.37** | **2.56** | **-2.42** |
| Carp-Free | -0.11 | -0.33 | 0.60 | **-3.67** | **1.78** | 0.60 | **-3.29** |
| Carp-Par | **-2.27** | **1.27** | 1.00 | **-3.17** | **1.55** | **1.14** | **-6.82** |
| Carp-ParCon | -0.11 | 0.09 | 0.00 | **-4.40** | **3.37** | **2.38** | **-2.77** |
| Punt-Free | -0.22 | -0.33 | 0.80 | **-4.67** | **2.87** | 0.50 | **-2.40** |
| Punt-Par | **-2.31** | **1.40** | **1.33** | **-4.17** | **2.18** | 1.00 | **-6.29** |
| Punt-ParCon | 0.00 | -0.33 | 0.40 | **-5.60** | **4.25** | **2.10** | **-2.69** |
| Flens-Free | -0.43 | 0.00 | 0.67 | -0.50 | 0.64 | **-2.09** | -0.20 |
| Flens-Par | **-2.45** | **1.33** | **1.67** | -0.17 | **-1.29** | **1.20** | **-4.86** |
| Flens-ParCon | 0.50 | **-1.33** | **1.20** | -0.60 | -0.17 | **2.00** | **-5.50** |
| Otag-Free | **-2.22** | 1.00 | **1.80** | **-6.00** | **2.11** | 0.75 | -0.83 |
| Otag-Par | -0.82 | -0.57 | **2.00** | **-4.67** | **1.67** | 0.80 | **-6.75** |
| Otag-ParCon | -0.62 | -0.67 | **1.80** | **-5.60** | **2.00** | **1.33** | **-6.56** |
| Sylt-Free | **-2.00** | 0.77 | **1.80** | 0.25 | **-1.71** | 0.80 | 0.20 |
| Sylt-Par | **-1.40** | 0.23 | **1.57** | 0.20 | **-1.29** | 1.00 | **-6.20** |
| Sylt-ParCon | -0.70 | 0.00 | **1.20** | 0.67 | -0.75 | **2.00** | **-4.29** |
| Ythan-Free | **-3.12** | **1.18** | **2.00** | **-3.17** | 0.56 | 0.25 | 0.00 |
| Ythan-Par | **-3.42** | **1.33** | **3.00** | **-2.29** | -0.44 | 0.25 | -0.50 |
| Ythan-ParCon | -0.80 | -0.38 | **1.83** | **-4.60** | **1.11** | -0.40 | **-6.40** |
