# Supplementary material for: Parasites Affect Food Web Structure Primarily through Increased Diversity and Complexity
Source: PLoS Biol. 2013 Jun 11;11(6):e1001579. doi: 10.1371/journal.pbio.1001579 (PMC3679000; doi:10.1371/journal.pbio.1001579)
Supplement: Table S7 — Niche model errors for web structure properties. See Table S1 for food web naming conventions. The values show the niche MEs for properties related to types of species in the web. The properties are defined in Table 1 (Metrics 13–19). Values of ME>|1| are shown in bold and indicate a poor fit of the niche model prediction to the empirical value. Negative MEs indicate niche model underestimation of the empirical value; positive MEs indicate niche model overestimation of the empirical value. (DOCX) [file pbio.1001579.s014.docx]

**Table S7. Niche Model Errors for Web Structure Properties**

| Food Web-Type | LinkSD | GenSD | VulSD | TL | MaxSim | Path | Clus |
| --- | --- | --- | --- | --- | --- | --- | --- |
| Fals-Free | **-1.60** | 0.29 | **-2.38** | 0.45 | 0.13 | 0.57 | **1.06** |
| Fals-Par | **-2.85** | 0.95 | **-1.52** | -0.27 | 0.55 | -0.32 | 0.15 |
| Fals-ParCon | **-3.42** | 0.60 | -0.71 | -0.78 | 0.51 | **-2.04** | **1.73** |
| Carp-Free | **-1.42** | 0.95 | **-2.67** | 0.61 | 0.64 | -0.20 | 0.58 |
| Carp-Par | **-3.00** | **1.32** | **-1.50** | **-1.36** | **2.50** | **-1.93** | -0.42 |
| Carp-ParCon | **-2.98** | 0.97 | **-1.24** | **-2.22** | **2.20** | **-2.22** | **1.23** |
| Punt-Free | **-1.44** | 0.81 | **-2.51** | 0.80 | 0.53 | -0.71 | 0.70 |
| Punt-Par | **-3.78** | **1.08** | **-1.58** | **-1.32** | **1.37** | **-1.90** | -0.75 |
| Punt-ParCon | **-2.92** | 0.16 | **-1.12** | **-1.54** | 0.78 | **-1.93** | **1.46** |
| Flens-Free | 0.66 | -1.00 | -0.35 | **1.25** | -0.22 | **1.28** | 1.00 |
| Flens-Par | **-2.55** | 0.91 | **-2.41** | -0.80 | 0.32 | -0.84 | 0.53 |
| Flens-ParCon | **-3.56** | **-1.36** | -0.65 | -0.81 | -0.46 | -0.68 | 0.06 |
| Otag-Free | -0.14 | **-3.39** | **-2.50** | 0.73 | **-1.45** | 0.18 | 0.38 |
| Otag-Par | **-2.22** | 0.54 | **-2.49** | -0.19 | 0.51 | 0.03 | -0.32 |
| Otag-ParCon | **-2.57** | 0.14 | **-2.46** | -0.37 | 0.06 | -0.25 | -0.06 |
| Sylt-Free | 0.40 | **-4.68** | **-2.89** | 0.30 | 0.21 | -0.41 | -0.57 |
| Sylt-Par | **-2.55** | **1.24** | **-2.34** | -0.39 | **1.14** | -0.15 | -0.44 |
| Sylt-ParCon | **-3.49** | 0.76 | **-2.34** | **-1.25** | **1.61** | **-1.12** | 0.18 |
| Ythan-Free | **-2.97** | 0.38 | **-4.64** | -0.36 | **2.24** | **1.03** | -0.79 |
| Ythan-Par | **-3.70** | **1.09** | **-5.68** | **-1.42** | **3.38** | **1.11** | **-1.79** |
| Ythan-ParCon | **-2.05** | 0.54 | **-2.95** | **-1.76** | **3.44** | -0.86 | -0.53 |
