# Supplementary material for: Parasites Affect Food Web Structure Primarily through Increased Diversity and Complexity
Source: PLoS Biol. 2013 Jun 11;11(6):e1001579. doi: 10.1371/journal.pbio.1001579 (PMC3679000; doi:10.1371/journal.pbio.1001579)
Supplement: Table S8 — Probabilistic niche model results. See Table S1 for food web naming conventions. f L-1D and f L-2D indicate the fraction of links in an empirical web predicted correctly by the one-dimensional and two-dimensional versions of the probabilistic niche model (Box 1), respectively. AIC-1D and AIC-2D give the Akaike Information Criterion values [93] for the performance of the one-dimensional and two-dimensional versions of the probabilistic niche model. (DOCX) [file pbio.1001579.s015.docx]

**Table S8. Probabilistic Niche Model Results**

| Food Web-Type | *f_L-1D_* | *f_L-2D_* | AIC-1D | AIC-2D |
| --- | --- | --- | --- | --- |
| Fals-Free | 0.665 | 0.838 | 1525 | 1229 |
| Fals-Par | 0.593 | 0.774 | 5419 | 3860 |
| Fals-ParCon | 0.657 | 0.795 | 7364 | 5189 |
| Carp-Free | 0.633 | 0.795 | 2270 | 1828 |
| Carp-Par | 0.558 | 0.728 | 6484 | 4882 |
| Carp-ParCon | 0.578 | 0.735 | 9840 | 6857 |
| Punt-Free | 0.656 | 0.809 | 3000 | 2253 |
| Punt-Par | 0.561 | 0.747 | 9286 | 6362 |
| Punt-ParCon | 0.576 | 0.754 | 13595 | 9038 |
| Flens-Free | 0.756 | 0.927 | 863 | 701 |
| Flens-Par | 0.631 | 0.817 | 2619 | 1955 |
| Flens-ParCon | 0.645 | 0.831 | 3551 | 2403 |
| Otag-Free | 0.657 | 0.819 | 2125 | 1687 |
| Otag-Par | 0.588 | 0.749 | 3604 | 2719 |
| Otag-ParCon | 0.566 | 0.764 | 4462 | 3267 |
| Sylt-Free | 0.601 | 0.745 | 3217 | 2794 |
| Sylt-Par | 0.593 | 0.719 | 5443 | 4518 |
| Sylt-ParCon | 0.596 | 0.730 | 7792 | 6110 |
| Ythan-Free | 0.609 | 0.677 | 1346 | 1447 |
| Ythan-Par | 0.516 | 0.624 | 2454 | 2396 |
| Ythan-ParCon | 0.555 | 0.693 | 4348 | 3477 |
